# Region-Specific and Age-Dependent Multitarget Effects of Acetylcholinesterase Inhibitor Tacrine on Comprehensive Neurotransmitter Systems

**DOI:** 10.1021/acschembio.1c00803

**Published:** 2021-12-21

**Authors:** Elva Fridjonsdottir, Theodosia Vallianatou, Ioannis Mantas, Reza Shariatgorji, Anna Nilsson, Luke S. Schembri, Luke R. Odell, Per Svenningsson, Per E. Andrén

**Affiliations:** † Department of Pharmaceutical Biosciences, Medical Mass Spectrometry Imaging, Uppsala University, SE-75124 Uppsala, Sweden; ‡ Department of Clinical Neuroscience, Section of Neurology, Karolinska Institutet, SE-17177 Stockholm, Sweden; § Science for Life Laboratory, Spatial Mass Spectrometry, Uppsala University, SE-75124 Uppsala, Sweden; ∥ Department of Medicinal Chemistry, Uppsala University, SE-75123 Uppsala, Sweden

## Abstract

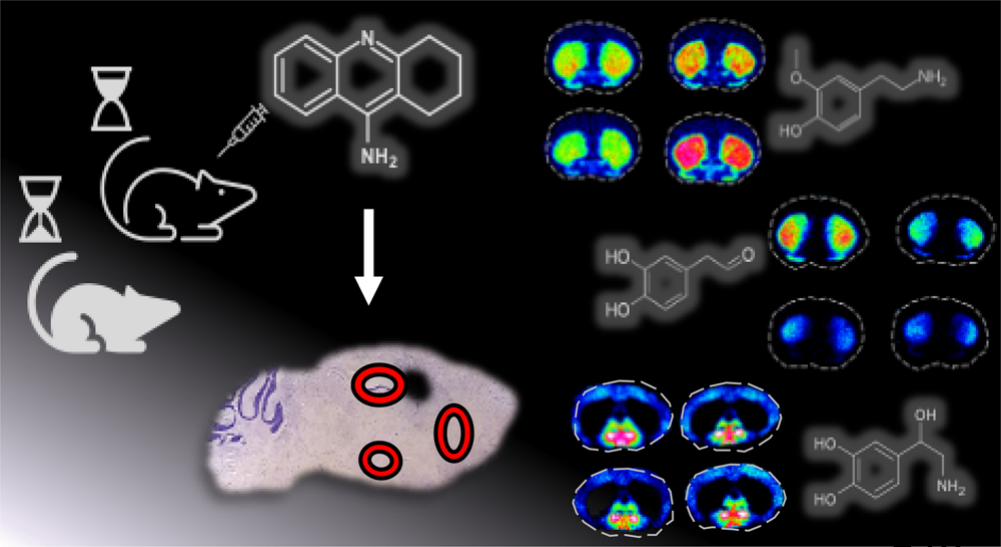

Regional brain distribution and metabolism of neurotransmitters and their response to drug treatment are fundamentally important for understanding the central effects of neuroactive substances. We used matrix-assisted laser desorption/ionization mass spectrometry imaging in combination with multivariate analysis to visualize in anatomical detail metabolic effects of aging and tacrine-mediated acetylcholinesterase inhibition on comprehensive neurotransmitter systems in multiple mouse brain regions of 12-week-old and 14-month-old mice. We detected age-related increases in 3,4-dihydroxyphenylacetaldehyde and histamine, indicating oxidative stress and aging deficits in astrocytes. Tacrine had a significant impact on the metabolism of neurotransmitters in both age groups; predominantly, there was an increased norepinephrine turnover throughout the brain and decreased 3-methoxy tyramine, a marker for dopamine release, in the striatum. The striatal levels of histamine were only elevated after tacrine administration in the older animals. Our results demonstrated that tacrine is a multitarget and region-specific neuroactive agent, inducing age-specific responses. Although well-studied, the complete mechanisms of the action of tacrine are not fully understood, and the current findings reveal features that may help explain its treatment-related effectiveness and central side effects.

## Introduction

Progressive failure of defined neurotransmitter systems is involved in major neurodegenerative disorders, such as Alzheimer’s disease (AD) and Parkinson’s disease.^1^ Because aging is the primary risk factor for the onset of these diseases, an increasing number of studies have been conducted to detect changes associated with normal (nonpathological) aging, including in neurotransmitters,^2^ transporters,^3,4^ receptors,^5,6^ and metabolizing enzymes.^7,8^

Treatment through acetylcholinesterase (AChE) inhibition is one of only a few therapies with proven clinical utility for the treatment of dementia and AD.^9^ Targeting the cholinergic system can in turn modulate monoaminergic systems through nicotinic and muscarinic cholinergic receptors located on monoaminergic neurons.^10−12^ Therefore, manipulation of the cholinergic system may influence the signaling of all other monoaminergic systems, and age-induced changes in neurotransmitter systems may affect the response to such pharmacological interventions.^13^

We recently showed that normal aging reduces the responsivity of the cholinergic system to the AChE inhibitor tacrine in the retrosplenial cortex (RSC).^14^ In addition to being an AChE inhibitor, tacrine has been shown to have other effects on the cholinergic system, such as increasing the synthesis and release of acetylcholine (ACh) and regulating the muscarinic and nicotinic receptors.^15^ Furthermore, tacrine has been reported to interact with the monoaminergic systems. Several studies have highlighted the potential complexity of the mechanisms responsible for the memory-enhancing properties of tacrine.^16−22^ In particular, it has been reported to have a wide range of targets, such as the γ-aminobutyric acid (GABA)ergic, nitrinergic, and glutamatergic pathways, in addition to AChE.^15^ Age-related alterations in the brain regional content of specific neurotransmitters are an additional factor that complicates the responsiveness to tacrine treatment.^14,23^

Matrix-assisted laser desorption/ionization mass spectrometry imaging (MALDI-MSI) has emerged as a useful technique for the molecular-specific imaging of neurochemicals directly in brain tissue sections.^24^ It is a powerful ex vivo visualization method for quantifying the abundance and lateral distribution of endogenous metabolites, lipids, peptides, and small proteins, as well as pharmaceutical compounds.^25^

We employed an innovative molecular-specific method using Fourier-transform ion cyclotron resonance (FTICR) MALDI-MSI, which enables the simultaneous, direct mapping of comprehensive neurotransmitter metabolic pathways, including dopamine (DA), norepinephrine (NE), serotonin (5-HT), GABA, and histamine (His) systems to investigate the effect of the well-studied, but complex and not fully understood, AChE inhibitor tacrine in mice of two different ages: 12-week-old (12-w) and 14-month-old (14-m). We showed that DA metabolites were significantly altered by tacrine in regions highly innervated by DA neurons, such as caudate-putamen (CPu). In the same region, an age-specific increase in oxidative DA metabolism was observed, and NE metabolism in cortical areas was found to be highly dependent on age and tacrine administration. By studying these metabolic pathways and considering the significance of each metabolite,^26,27^ spatial information was obtained about the neuronal activity and turnover in multiple brain regions.

## Results

### Neurotransmitter and Metabolite Identification

The identification of the neurotransmitters and their metabolites analyzed in this study (Figure S1) was based on the ultrahigh mass accuracy provided by FTICR MS, which achieved mass errors below ±1.2 ppm (Table S1) for all the identified analytes.^28^ Tandem MS (MS/MS) spectra were acquired from tissue sections and compared with authentic standards to corroborate the identification of DA, NE, 3,4-dihydroxyphenylglycol (DOPEG), 3-methoxy tyramine (3-MT), 5-HT, and 5-hydroxyindoleacetic acid (5-HIAA) (Figure S2).

### Visualization of Multiple Neurotransmitter Systems in a Mouse Brain Tissue Section

Imaging of the major neurotransmitters, that is, DA, 5-HT, NE, and GABA, in a sagittal section provided an overview of brain regions important for each investigated neurotransmitter system, which were annotated on the same Nissl-stained section (Figure S3a). The results clearly demonstrated the dopaminergic nigrostriatal pathway from the substantia nigra pars compacta (SNc) to the CPu^32^ (Figure S3b). DA detected in the nucleus accumbens (Acb) and tubercle (Tu) (Figure S3b) was attributed to the mesolimbic pathway.^33^ A dense serotonergic input to the substantia nigra pars reticulata (SNr) was observed. 5-HT was abundant in the bed nucleus of the stria terminalis (BNST), hypothalamus (Hyp), hippocampus (Hip), and Tu but was less abundant in the cortex and CPu (Figure S3c). High NE levels were detected in the locus coeruleus (LC), the major NE-providing nucleus to the forebrain,^34^ BNST, and Hyp, but lower levels were found in the Hip, Th, and cortex regions (Figure S3d). High levels of GABA were detected across the whole brain section, but it was particularly abundant in the SNr, BNST, and Hyp (Figure S3e).

### Unsupervised Analysis Showed That Tacrine Had a Major Impact on Neurotransmitter Metabolism

**Figure 1:**
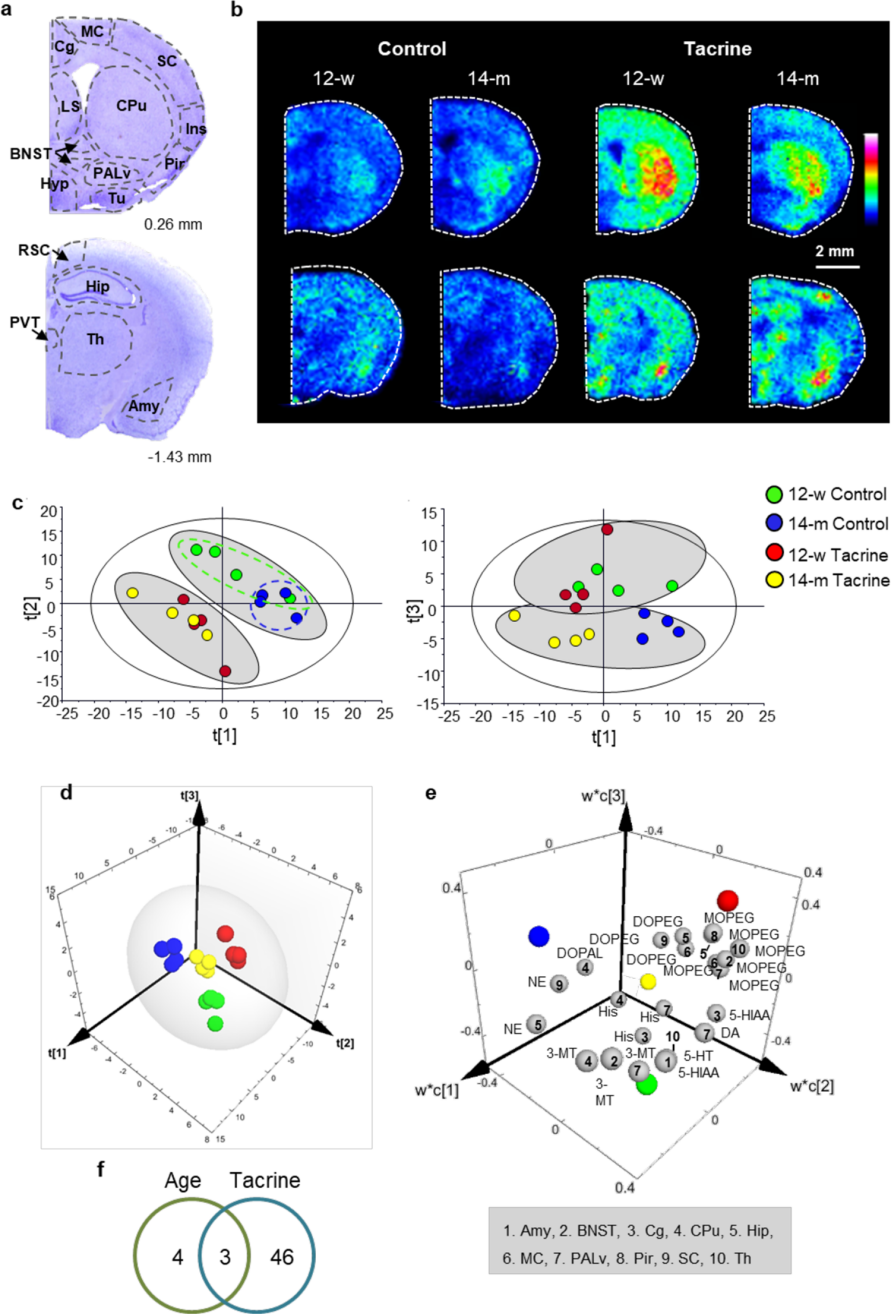
Multivariate exploration of MALDI-MSI data. a, Nissl-stained coronal sections from a 14-m control animal at 0.26 mm and −1.43 mm from bregma with the analyzed brain regions annotated. b, MALDI-MS images of acetylcholine from levels 0.26 mm (upper panel) and −1.43 mm (lower panel) from bregma. Images are shown using a rainbow scale, indicating the relative abundance of acetylcholine scaled from 0 to 70%. The lateral resolution is 100 μm. c, Score plots of the first and second components of the PCA model (left) and the first and third components of the PCA model (right). Objects are colored according to age (12-w or 14-m) and treatment (tacrine or control). Gray ovals show grouping of samples according to treatment and age. d, 3D score plot of the three-principal-component PLS-DA model. Objects are colored according to age (12-w or 14-m) and treatment (tacrine or control). e, 3D loading plot of the three-component PLS-DA model. The colored spheres represent the center of each group. The gray spheres represent the significant neurotransmitters and metabolites in specific brain areas annotated by numbering. f, Diagram showing the results of significant alterations in neurotransmitters and metabolites related to age, tacrine, or both from two-way ANOVA with the FDR correction. Abbreviations: 12-w, 12-week-old; 14-m, 14-month-old; DA, dopamine; 3-MT, 3-methoxy tyramine; DOPAL, 3,4-dihydroxyphenylacetaldehyde; NE, norepinephrine; DOPEG, 3,4-dihydroxyphenylglycol; MOPEG, 3-methoxy-4-hydroxyphenylglycol; 5-HT, 5-hydroxytryptamine; 5-HIAA, 5-hydroxyindoleacetic acid; His, histamine; Amy, amygdala; BNST, bed nucleus of the stria terminalis; Cg, cingulate cortex; CPu, caudate-putamen; Hip, hippocampus; Hyp, hypothalamus; Ins, insular cortex; LS, lateral septum; MC, primary and secondary motor cortex; PALv, ventral pallidum; Pir, piriform cortex; PVT, periventricular thalamic nucleus; RSC, retrosplenial cortex; SC, primary and secondary somatosensory cortex; Th, thalamus; Tu, tubercle.

Neurotransmitter and metabolite abundances in 16 brain regions were included in the analyses to investigate the effect of age (12-w and 14-m) and tacrine (control and tacrine-administered animals)^29^ (Figure 1a). MALDI-MSI at both brain levels, that is, 0.26 mm and −1.43 mm from bregma, showed an increase in acetylcholine in the tacrine-administered animals (Figure 1b). First, unsupervised PCA was performed by including data for the 13 identified neurotransmitters and neurotransmitter metabolites. PCA score plots showed relationships between the individual samples based on the neurotransmitters and their metabolites (Figure 1c). The score plot of the first (*t*[1]) and second (*t*[2]) components, explaining 25.8 and 18.9% of the total variance, respectively, demonstrated an absence of strong outliers and revealed treatment-related clustering of the data (gray ovals in Figure 1c). This led to the initial observation that tacrine had major effects on the neurotransmitters and their metabolites. However, the control groups were separated from the tacrine-administered groups according to different components, (14-m by *t*[1] and 12-w by *t*[2] Figure 1c), reflecting an age-associated effect of tacrine. The scores of the third component (*t*[3]), explaining 10.8% of the total variance, demonstrated clear separation according to age (*P* < 0.05) when plotted against *t*[1], with the 14-m samples having negative *t*[3] values (Figure 1c). Further information was obtained by the detailed analysis of the loading of each metabolite in each brain region, which revealed specific trends, for example, the brain regions CPu, Hyp, insular cortex (Ins), ventral pallidum (PALv), piriform cortex (Pir), and somatosensory cortex (SC) showed a general trend of increased metabolite levels in both tacrine-administered groups compared to the 14-m control group (Figure S4). In addition, the CPu, PALv, and Tu had reduced neurotransmitter and metabolite levels after tacrine administration, whereas the opposite trend was observed for the primary and secondary motor cortex (MC) and periventricular thalamic nucleus (PVT, Figure S4). The amyglada (Amy), MC, RSC, and thalamus (Th) had increased overall metabolite levels in the 12-w compared to 14-m samples (Figure S4).

### Supervised Partial Least Squares-Discriminant Analysis Determined Locations of Major Metabolite Differences between the Four Groups

Four classes of samples were defined, that is, from 12-w and 14-m, saline or tacrine-administered animals, as the response variables. Following optimization, a model with three principal components and high apparent validity (*R*^2^ = 0.909, *Q*^2^ = 0.833) was obtained. The separation of these groups by the model is illustrated in the three-dimensional (3D) score plot in Figure 1d. The significance of their separation was confirmed by the two-way analysis of variance (ANOVA) (with Tukey’s post hoc test, Table S4). The first component significantly separated samples according to the treatment (*P* < 0.001), whereas the second and third components separated them according to age (*P* < 0.01). The three-dimensional (3D) loading plot showed the correlation of significant molecules, that is, neurotransmitters and metabolites in particular brain areas, to the four different groups, revealing associations between specific neurotransmitters and metabolites in particular brain areas (Figure 1e). The results from the partial least squares-discriminant analysis (PLS-DA) were in accordance with the overall trends observed in the original PCA but provided a more specific and detailed description of regional alterations of multiple metabolites. The statistical significance of the results was further evaluated by two-way ANOVA with false discovery rate (FDR) correction (Table S5). We confirmed 56 significant changes, 46 of which were related to tacrine, four were related to age, and three were related to both age and tacrine (Figure 1f). The changes are described in detail below with respect to each neurotransmitter system.

### Changes in DA Metabolism

**Figure 2:**
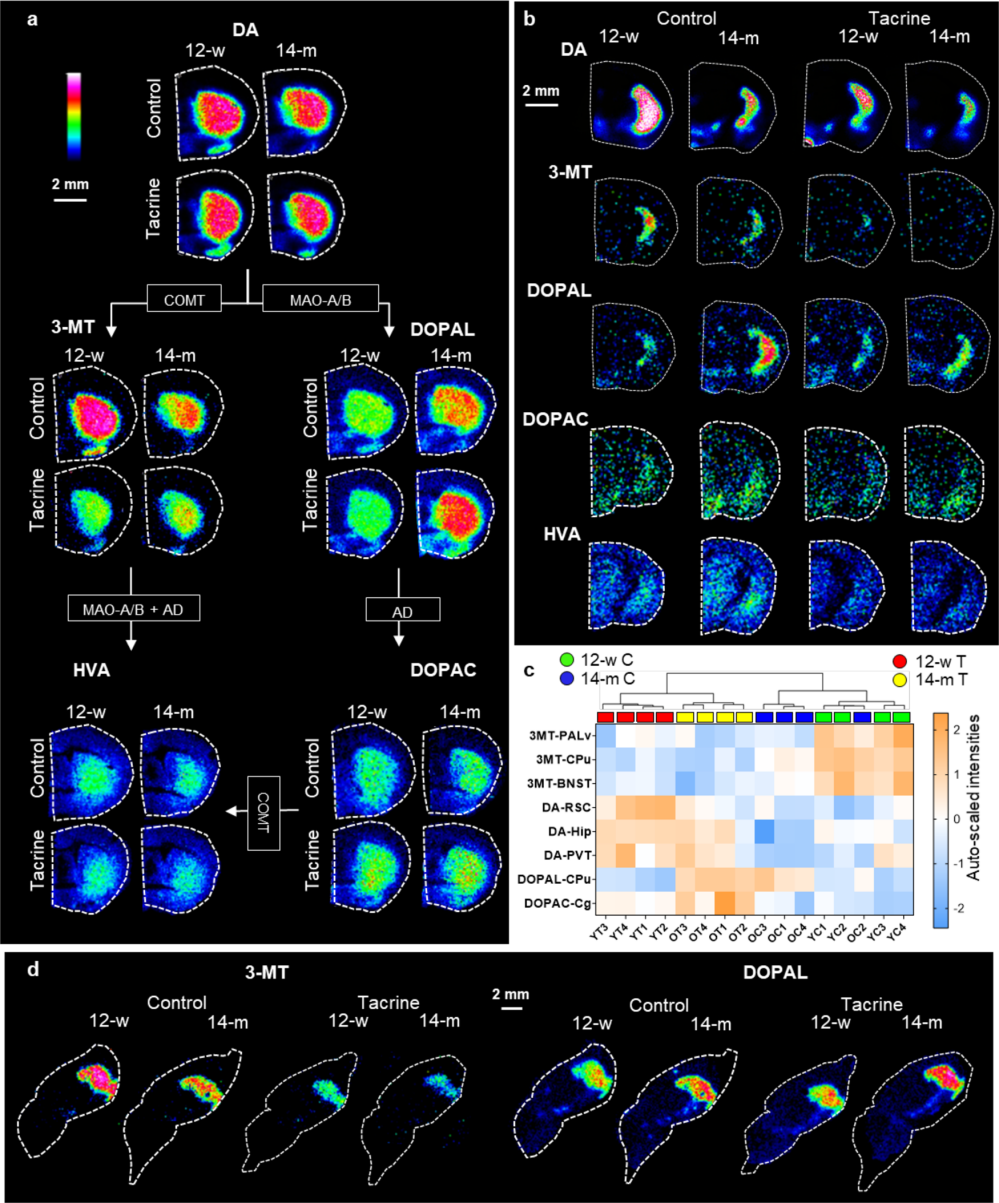
MALDI-MS images showing age- and tacrine-related alteration of the dopaminergic metabolic pathways in mouse brain tissue sections. a, Metabolic pathway of DA representative tissue sections (at 0.26 from bregma) from each studied group. The lateral resolution is 100 μm. The enzymes involved are annotated in boxes. b, MALDI-MS images of dopamine metabolites (at −1.43 mm from bregma) from each studied group with a lateral resolution of 50 μm. c, Heatmap based on the autoscaled log intensities generated for the selected top eight (ANOVA, *P* < 0.05) features. d, MALDI-MS images of 3-MT and DOPAL from each studied group in sagittal sections at 1.2 mm lateral from the midline with a lateral resolution of 100 μm. For a, b, and d, the ion intensity rainbow color scales are according to optimal visualization. Abbreviations: 12-w, 12-week-old; 14-m, 14-month-old; AD, aldehyde dehydrogenase; COMT, catechol-*O*-methyltransferase; MAO, monoamine oxidase, DA, dopamine; 3-MT, 3-methoxy tyramine; DOPAL, 3,4-dihydroxyphenylacetaldehyde; DOPAC, 3,4-dihydroxyphenylacetic acid; HVA, homovanillic acid; BNST, bed nucleus of the stria terminalis; Cg, cingulate cortex; CPu, caudate-putamen; Hip, hippocampus; PVT, periventricular thalamic nucleus; RSC, retrosplenial cortex.

DA and four downstream metabolites formed by catechol-*O*-methyltransferase (COMT) and monoamine oxidase (MAO) in different sequences, that is, 3-MT, DOPAL, 3,4-dihydroxyphenylacetic acid (DOPAC), and homovanillic acid (HVA), were imaged by MALDI-MSI (Figure 2a,b). Tacrine administration significantly increased DA in the Hip, PVT, and RSC (Table S5) for both age groups. DOPAC was elevated by tacrine in the target structures of the mesolimbic and mesocortical pathways, that is, the PALv, BNST, Hyp, cingulate cortex (Cg), Ins, and Th but not the CPu (Table S5). However, 3-MT was decreased in many structures following tacrine treatment, including the CPu, BNST, PALv, and Amy (Table S5). 3,4-Dihydroxyphenylacetaldehyde (DOPAL) levels were significantly elevated in the CPu of the older animals (Table S5). There was a trend for 3-MT levels to be decreased by aging in the CPu, PALv, and BNST (Figures 1e and 2a,c). There were no significant effects on HVA related to tacrine or age. Hierarchical clustering of samples based on the top eight significant metabolites showed clustering of the samples according to the different groups (Figure 2c). The tacrine-related decrease in 3-MT and age-related increase in DOPAL were detected in the sagittal sections of each group (Figure 2d).

### Changes in the Histaminergic System

**Figure 3:**
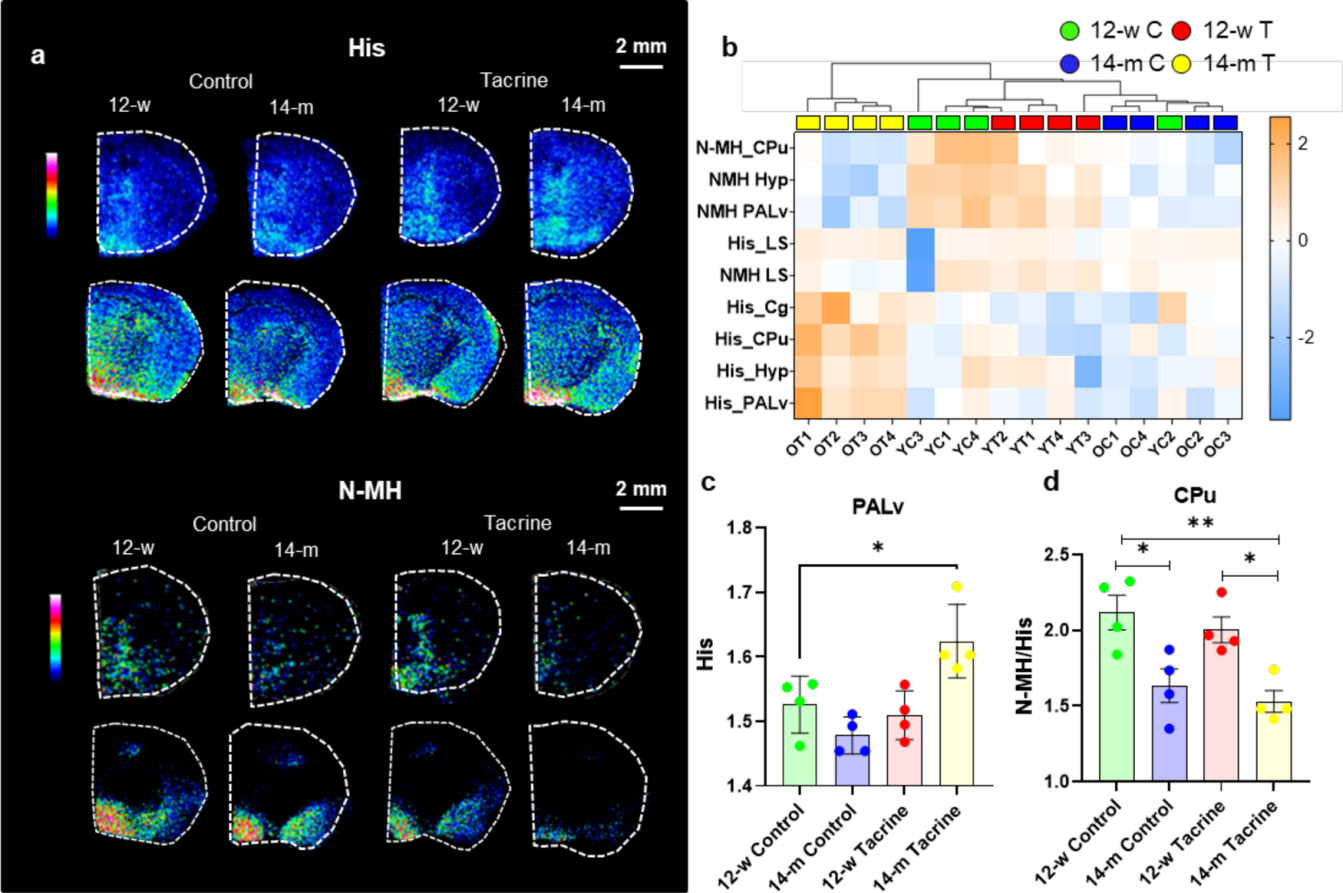
Histaminergic metabolic changes associated with aging and tacrine. a, MALDI-MS images of His and N-MH at coronal brain levels 0.20 mm from bregma (upper panels) and −1.43 mm from bregma (lower panels). The lateral resolution is 100 μm for images at level 0.20 mm and 50 for images at level −1.43. The ion intensity rainbow color scales are scaled according to the optimal visualization of each metabolite. b, Heatmap based on the auto-scaled log intensities, generated for the selected top nine (ANOVA, *P* < 0.05) features. c, Comparison of His levels (log ion intensity) in the PALv between each experimental group using one-way ANOVA with Tukey’s post hoc test. d, Comparison of the N-MH/His ratio in the CPu between each experimental group using one-way ANOVA with Tukey’s post hoc test. Means and standard deviation are shown. *P < 0.05, **P < 0.01. Abbreviations: 12-w, 12-week-old; 14-m, 14-month-old; Cg, cingulate cortex; CPu, caudate-putamen; N-MH, *N*-methylhistamine; His, histamine; Hyp, hypothalamus; LS, lateral septum; PALv, ventral pallidum.

MALDI-MS images revealed the distribution of His and low levels of its metabolite *N*-methylhistamine (N-MH) (Figure 3a). His was significantly increased in the CPu of 14-m animals compared to 12-w animals (Table S5). In addition, tacrine caused a significant increase in His in the PALv (Table S5). Levels of the His metabolite N-MH were low, but MALDI-MS images indicated a slight decrease in its levels in older animals of both treatment groups in His-rich regions (Figure 3b). Interestingly, the histaminergic system demonstrated an age-related response to tacrine, as its administration increased His levels in the Cg, CPu, and PALv only in the 14-m animals (Figures 1e and 3b,c, Table S5). The N-MH/His ratio was significantly higher in the 12-w animals than in the 14-m animals regardless of tacrine treatment (Figure 3d).

### Changes in Norepinephrine Metabolism

**Figure 4:**
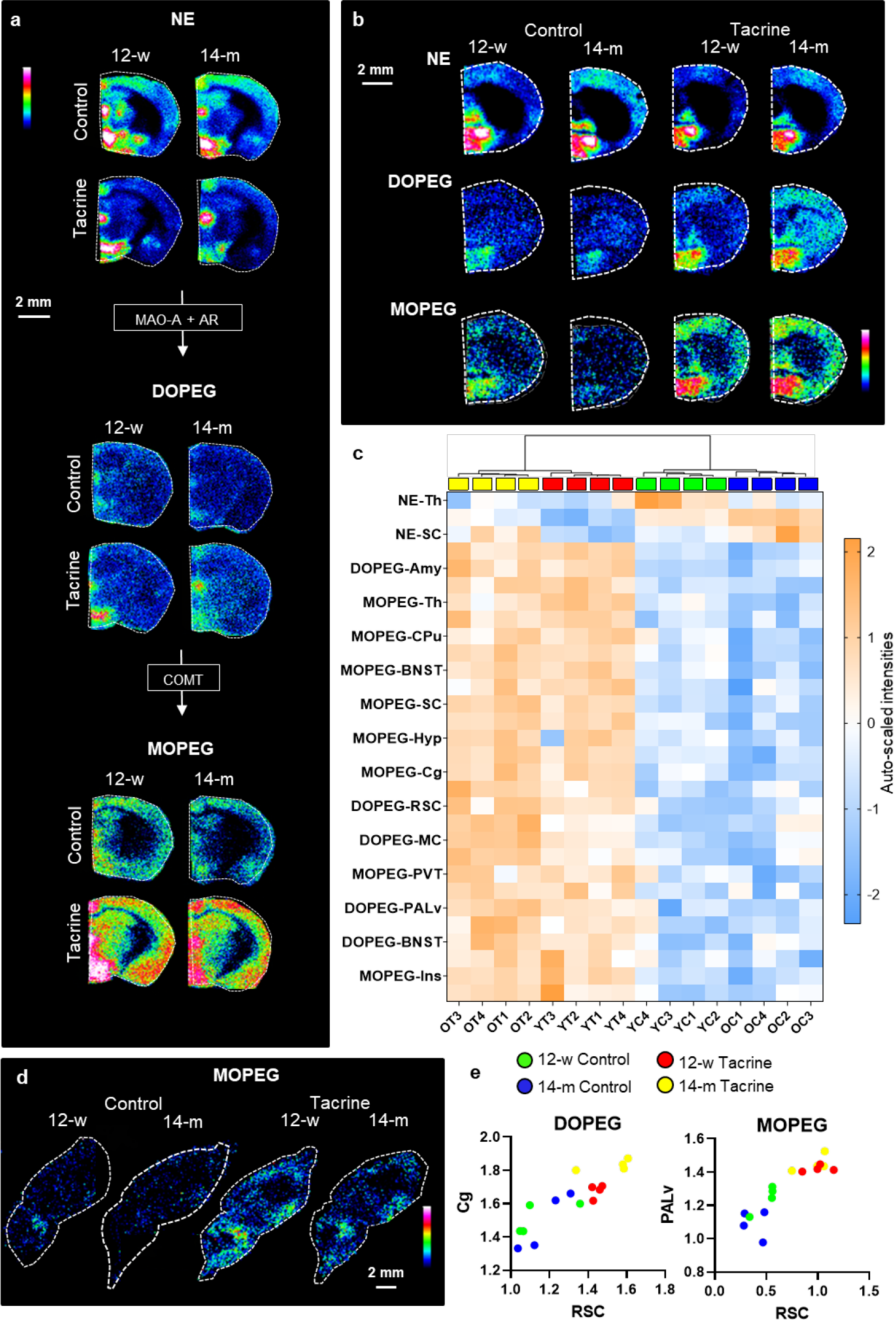
Changes in NE metabolism in response to age and tacrine administration. a, MALDI-MS images of NE metabolic pathway from coronal mouse brain tissue sections (bregma −1.43 mm) at a lateral resolution of 50 μm. b, MALDI-MS images of NE and related metabolites (bregma 1.26) at lateral resolution of 100 μm. c, Heatmap based on the auto-scaled log intensities generated for the top 30 (ANOVA, *P* < 0.05) features. d, MALDI-MS images of DOPEG and MOPEG from each studied group in sagittal sections at 1.2 mm lateral from the midline at a lateral resolution of 100 μm. e, Correlation (Pearson coefficient *r*) of RSC levels of DOPEG and MOPEG with the corresponding levels in Cg and PALv, respectively. Abbreviations: 12-w, 12-week-old; 14-m, 14-month-old; AR, aldehyde reductase; COMT, catechol-*O*-methyltransferase; MAO-A, monoamine oxidase A; DOPEG, 3,4-dihydroxyphenylglycol; NE, norepinephrine; MOPEG, 3-methoxy-4-hydroxyphenylglycol; Amy, amygdala; BNST, bed nucleus of the stria terminalis; Cg, cingulate cortex; CPu, caudate-putamen; Hyp, hypothalamus; Ins, insular cortex; MC, primary and secondary motor cortex; PALv, ventral pallidum; PVT, paraventricular thalamic nucleus; RSC, retrosplenial cortex; SC, primary and secondary somatosensory cortex; Th, thalamus.

MALDI-MS images of NE, the metabolites DOPEG and, further downstream, 3-methoxy-4-hydroxyphenylglycol (MOPEG) showed that tacrine administration caused a substantial elevation of the NE metabolites in multiple regions (Figure 4a,b) for both age groups. NE levels were decreased in the SC, Hip, Th, and Amy, following tacrine administration, DOPEG was significantly increased in all analyzed brain regions, except the lateral septum (LS), CPu, Hyp, and Tu, and MOPEG was significantly increased in all analyzed brain regions (Figure 4a,b and Table S5). DOPEG levels in the SC were significantly elevated in the 14-m mice (Figure 4a, Table S5). In addition, the 14-m mice had significantly lower levels of MOPEG in the Th than the 12-w mice regardless of tacrine administration (Figure 4a, Table S5). The brain regional metabolic profile of NE was strongly related with aging and tacrine treatment, as shown by the clustering of samples from the same groups (Figure 4c). MALDI images of MOPEG in sagittal mouse tissue sections demonstrated the increase in MOPEG in the tacrine-treated animals (Figure 4d). Based on our previous findings regarding the age-dependent response of the cholinergic system in the RSC,^14^ we explored the correlations of NE metabolites in this region (Figure 4e). While the RSC levels of DOPEG were most significantly correlated with the corresponding levels of the metabolite in the Cg, which is anatomically connected with the RSC,^35^ the highest correlation for MOPEG was detected between the PALv.

### Changes in Serotonin and GABA Metabolism

MALDI-MS images of 5-HT and its metabolites 5-hydroxyindoleacetaldehyde (5-HIAL) and 5-HIAA are shown in Figure S5. We found that tacrine increased 5-HIAA in the Cg and MC (Table S5). 5-HIAA levels were decreased in the Amy with age (Table S5). In addition, PLS-DA results suggested that 5-HT in the Th in 12-w control animals was higher than in the 14-m controls in both tacrine-treated groups, indicating that the 14-m animals had low levels of 5-HT that were not affected by tacrine (Figure 1f, Table S5). GABA was detected in the whole brain, with the highest levels in basal forebrain regions (Figure 4c). We did not detect any tacrine- or age-related changes in the levels of GABA.

### Imaging of Monoaminergic Brain Metabolism

**Figure 5:**
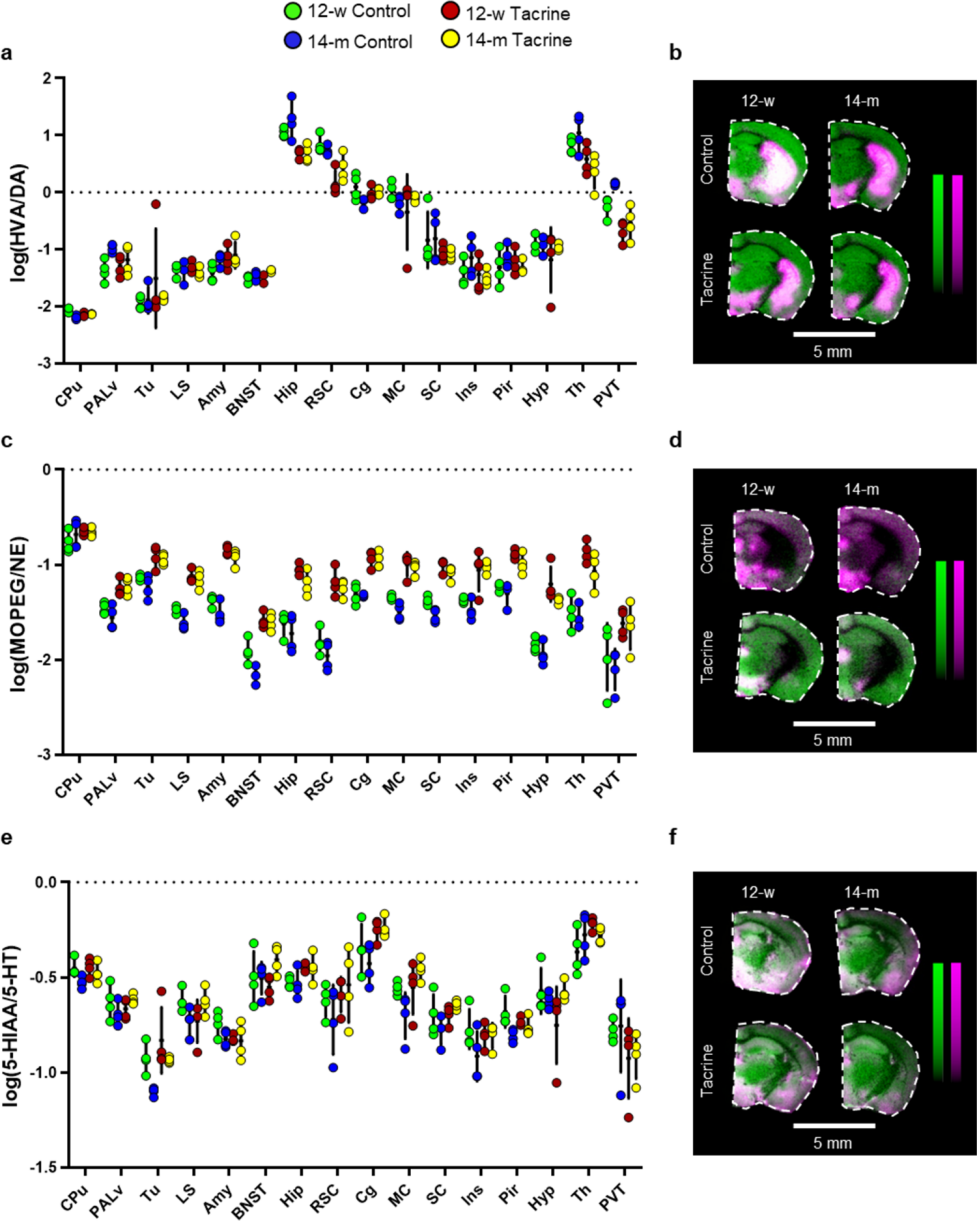
Effect of age and tacrine administration on regional monoaminergic metabolism. a, Dot plot of the log-transformed ratio between the RMS-normalized ion intensity of HVA and DA in multiple brain regions of the investigated groups. b, Overlaid MALDI-MS images of DA (magenta) and HVA (green) in coronal mouse brain tissue sections (−1.1 to −1.6 mm from bregma) of the four investigated groups. c, Dot plot of the log-transformed ratio between the RMS-normalized ion intensity of MOPEG and NE in multiple brain regions of the investigated groups. d, Overlaid MALDI-MS images of NE (magenta) and MOPEG (green) in coronal mouse brain tissue sections (−1.1 to −1.6 mm from bregma) of the four investigated groups. e, Dot plot of the log-transformed ratio between the RMS-normalized ion intensity of 5-HIAA and 5-HT in multiple brain regions of the investigated groups. f, Overlaid MALDI-MS images of 5-HT (magenta) and 5-HIAA (green) in coronal mouse brain tissue sections (−1.1 to −1.6 mm from bregma) of the four investigated groups. Abbreviations: 12-w, 12-week-old; 14-m, 14-month-old; DA, dopamine; HVA,; homovanillic acid; NE, norepinephrine; MOPEG, 3-methoxy-4-hydroxyphenylglycol; 5-HT, 5-hydroxytryptamine; 5-HIAA, 5-hydroxyindoleacetic acid; Amy, amygdala; BNST, bed nucleus of the stria terminalis; Cg, cingulate cortex; CPu, caudate-putamen; Hip, hippocampus; Hyp, hypothalamus; Ins, insular cortex; LS, lateral septum; MC, primary and secondary motor cortex; Tu, olfactory tubercle; PALv, ventral pallidum; Pir, piriform cortex; PVT, paraventricular thalamic nucleus; RSC, retrosplenial cortex; SC, primary and secondary somatosensory cortex; Th, thalamus.

The turnover ratios HVA/DA, MOPEG/NE, and 5-HIAA/5-HT were used as indicators of monoaminergic metabolism (Figure 5). This approach provided a comparative snapshot analysis of monoaminergic metabolism among the investigated groups and brain regions. In addition, it aided the detection of brain areas that were particularly metabolically responsive to aging and tacrine.

In the case of DA metabolism, the HVA/DA ratio was lower in brain regions with dense afferent DA projections, such as the CPu and Tu (Figure 5a), highlighting the dominant role of dopaminergic innervation in these areas. Age-induced elevation of the HVA/DA ratio was observed in the control samples in the PALv, Th, and especially, PVT (Figure 5a). A tacrine-induced decrease in the turnover ratio was observed in the Hip, RSC, Th, and PVT (Figure 5a). The overlaid MS images of DA and HVA in coronal mouse brain tissue sections (−1.4 mm from bregma) also showed the relative distribution of these two molecules, further elucidating the turnover ratio analysis (Figure 5b). The MOPEG/NE turnover ratio was significantly increased after tacrine administration in most investigated areas, except the CPu, whereas an age-induced decline was observed in the BNST and SC of the control samples (Figure 5c, Table S6). A tacrine-induced increase in NE metabolism was also apparent in the overlaid MS images of NE and MOPEG (Figure 5d). Treatment with tacrine led to the age-specific elevation of the 5-HIAA/5-HT ratio in the Cg, MC, Tu, and RSC of 14-m animals (Figure 5e, Table S6). In Tu, the 5-HIAA/5-HT ratio was significantly lower in the 14-m control group compared to the 12-w control group (Figure 5e). 5-HT demonstrated different localization in subregions, such as hippocampal layers, compared to 5-HIAA (Figure 5f).

## Discussion

We applied MALDI-MSI to investigate changes in neurotransmitter levels and their metabolic pathways (dopaminergic, noradrenergic, serotonergic, histaminergic, and GABAergic) associated with normal aging and in response to AChE inhibition by tacrine. Our targeted imaging approach enabled simultaneous examination of multiple and comprehensive neurotransmitter metabolic pathways in specific brain regions and provided valuable information about their localization, abundance, and turnover ratio. Dominant patterns in the signaling systems induced by age and treatment, as well as inter-relations among the metabolites in the different brain areas, were observed.

Normal aging is characterized by transcriptional changes in astrocytes toward a proinflammatory profile.^36,37^ This proinflammatory state is concomitant with the elevation of astrocytic monoamine oxidase B (MAOB) and the subsequent generation of reactive oxygen species.^38^ Phenethylamine and *N*-methylhistamine are the major MAOB substrates, whereas 5-HT is metabolized by monoamine oxidase A (MAOA).^39^ DA, NE, tyramine, and tryptamine are catabolized by both isoenzymes.^39^

The current study showed a major aging-associated elevation of DOPAL in the CPu with simultaneous reduction of 3-MT and *N*-methylhistamine, indicating increased MAOB activity. Even though it has previously been reported that the levels of the MAOB enzyme increase with age,^40,41^ our study represents for the first time that actual changes have been depicted with anatomical detail. Because there is a global deterioration of astrocytic function, these changes were more prominent in regions where each metabolite was enriched. In addition, this is related to our previous findings regarding age-specific increase in antioxidant and neuroprotective metabolites, such as α-tocopherol, carnosine, and l-carnitine, in astrocytic dense brain regions, a possibly compensating mechanism for elevated oxidative brain activity.^42^ Another astrocytic enzyme that decreases with aging is histamine *N*-methyltransferase (HNMT).^7,43^ Accordingly, HNMT deficiency may contribute to the depletion of *N*-methylhistamine.^44^ Simultaneously, we observed an aging-correlated increase in His, which may further support the diminished HNMT action.^44^

Tacrine is a potent noncompetitive inhibitor of AChE that raises acetylcholine brain levels. Thus, tacrine’s cognitive effect may stem from the activation of the brain’s cholinergic receptors. However, it has been shown that tacrine displays several off-target actions, such as direct inhibition of nicotinic^41,45^ and muscarinic receptors.^46^

In the present study, we reported a novel and comprehensive analysis of the multitarget effects of tacrine in numerous neurotransmitter systems. The most striking tacrine effects were observed in MOPEG and 3-MT, which are COMT-derived metabolites of NE and DA, respectively.^27^ Both MOPEG and 3-MT have been suggested as indirect indicators for the release of their precursors.^27,47^ Consequently, we suggest that tacrine treatment resulted in higher NE and lower DA release, respectively. One explanation may be that the brainwide increase in acetylcholine by tacrine activates cholinergic receptors in multiple key brain regions that affect the tone of NE and DA neurons.^48,41,45^ MALDI-imaging of tacrine-treated brains has shown that acetylcholine levels are strikingly heightened in areas that display dense AChE staining, such as the CPu and Amy^14,29^ It is known that the medial central Amy nucleus projects to LC neurons.^49^ As a result, acetylcholine elevation may stimulate the Amy-LC pathway and enhance NE release. Regarding the DA system, tacrine treatment might stimulate CPu and Amy cells, which then either directly or indirectly inhibit DA neuron activity.^50^ Further investigations led by the present findings may indicate the exact mechanism of the effect of tacrine on these catecholamine systems.

We observed that aging caused higher NE in the Hip and SC, indicating that certain LC axonal projections are differentially affected by age. LC neurons exhibit aging vulnerability because they are involved in the neurodegenerative processes of Parkinson’s and Alzheimer’s disease.^51^ Tacrine administration normalized the NE content in Hip and SC to levels observed in adult mice. Hip atrophy is a key feature of the aging brain and is associated with not only cognitive and memory decline but also sleep disruption.^52,53^ In addition, Hip projects to RSC, a post-cingulate cortical area that has been associated with early onset of cognitive impairment.^54^ It was recently found that hippocampal efferents, specifically from the dorsal Hip to the RSC are required for memory acquisition.^55^ Therefore, there is a strong connection between the age-specific effect of tacrine on hippocampal NE levels and the opposite effect on ACh in the RSC, which we have previously reported.^14^ Furthermore, the Hip β_2_-adrenergic receptors are specifically localized in astrocyte-enriched areas, such as the stratum lacunosum moleculare.^56^ Given that aged Hip exhibits dysfunctional astrocytes and lactate shuttle impairment, tacrine administration may rescue these deficits by increasing NE release. As a result, apart from restoring aging cholinergic deficits, tacrine may rescue the Hip dysfunction through NE mechanisms.

Another age-related, region-specific, tacrine-induced change concerned His levels in the Cg, CPu, and PALv of 14-m animals, but not 12-w animals. Tacrine has been reported to behave as a potent inhibitor of HNMT in vitro.^57^ However, our results did not suggest the inhibition of HNMT in the younger animals, which would have been reflected by increased His levels. As shown in MALDI images, the highest His concentration was observed in the tuberomammillary nucleus of the hypothalamus, where the His-producing neurons are located.^58^ These cells send broad axonal projections to multiple forebrain regions, such as the Hip, cortex, Amy, and CPu.^58^ Similar to NE, His is involved in a broad range of brain functions, such as the sleep–wake cycle, memory, and cognition.^58^ As mentioned earlier, all these physiological functions decline during the aging process. Hence, tacrine-induced His changes may provide another aspect of tacrine benefits on aging-related deficits.

The extensive amount of information obtained from a single MALDI-MSI experiment can make it challenging to evaluate and filter out the most important and biologically relevant information. Here, we present a method to image, process, evaluate, and identify numerous neurotransmitter alterations, shedding light on the most affected neurotransmitters and brain regions under the biological conditions tested. Our study demonstrates the great potential of MALDI-MSI to investigate brain region-specific metabolite changes in neuropharmacology, normal aging, and neurological disorders.

In conclusion, the simultaneous and comprehensive imaging of multiple neurotransmitters and their metabolites can be used to evaluate the response of a multitarget psychoactive drug on neurotransmitter systems not targeted by the intended treatment. This is of high importance in an era when polypharmacology is widely explored in the multifactorial neuronal disorders. These effects are difficult to demonstrate with any other technique, but their identification is important to understand the pharmacodynamics, mechanism of action, and side effects of psychoactive drugs.

## Materials and Methods

### Chemicals

Water, methanol, and acetonitrile were of high-performance liquid chromatography (HPLC) grade (VWR, Stockholm, Sweden). The reactive matrix FMP-10 was synthesized in-house, as previously described.^28^ The deuterated analogues of acetylcholine (ACh-*d_9_*) and of α-cyano-4-hydroxycinnamic acid (CHCA-*d_4_*) were obtained from CDN Isotopes (Essex, UK), BOC Sciences (NY, USA), and Ubichem (Budapest, Hungary), respectively. All standards used for identifying analytes by MS/MS were purchased from Sigma-Aldrich (Stockholm, Sweden).

### Animal Experiment

Sixteen male mice (C57BL/6 J) of two ages (12-w and 14-m, obtained from Janvier labs, Scand-LAS Turku, Finland) were housed under controlled temperature and humidity (20 °C, 53% humidity) with 12 h light/12 h dark cycles and fed *ad libitum*. All experimental procedures were complied with European Council Directive 86/609/EEC and were approved by the local Animal Ethical Committee (approval nos. N40/13 and N275-15). Efforts were taken to minimize the number of animals used and their suffering. Tacrine was dissolved in saline and administered intraperitoneally (i.p.) at a dose of 10 mg/kg to both the 12-w and 14-m mice (*n* = 4 per group). Control animals were injected with an equivalent amount of vehicle (*n* = 4 per age group). Animals were decapitated 30 min after injection. Brains were rapidly dissected out, snap-frozen in dry-ice cooled isopentane, and stored at −80 °C to minimize postmortem degradation.

### Tissue Processing and Sample Preparation

Coronal brain tissue sections, 12 μm thick, were cut at −20 °C using a CM1900 UV cryostat-microtome (Leica Microsystems, Wetzlar, Germany) and subsequently thaw-mounted on conductive indium tin oxide-coated glass slides (Bruker Daltonics, Bremen, Germany). The interaural 4.06 mm (bregma 0.26 mm) brain level^29^ was selected as the most representative for investigating the dopaminergic system and the major monoaminergic brain pathways, that is, the BNST, Cg, CPu, Hyp, Ins, LS, primary and secondary MC, olfactory Tu, PALv, Pir, and primary and secondary SC. Sections at bregma −1.43 mm^29^ were collected to investigate the Amy, Hip, RSC, PVT, and Th. Tissue sections from coronal levels between bregma 0.26 mm and −1.6 mm were collected for additional analysis. Sagittal brain sections (level 1.2 mm)^29^ were collected to obtain an overview of the brain distribution of the monoaminergic neurotransmitters. The prepared slides were stored at −80 °C. Sections were desiccated at room temperature for 15 min prior to scanning on a flatbed scanner (Epson Perfection V500, Japan).

On-tissue derivatization was performed with the FMP-10 reactive matrix according to a previously described protocol.^28,30^ Briefly, a freshly prepared solution of FMP-10 (4.4 mM) in 70% acetonitrile was sprayed onto mouse brain tissue sections in 30 passes at 80 °C using a robotic sprayer (TM-Sprayer; HTX Technologies, Carrboro, NC) with a flow rate of 80 μL/min, spray head velocity of 1100 mm/min, 2.0 mm track spacing, and 6 psi nitrogen pressure.

Brain tissue preparation for MALDI-MSI of ACh was performed, as previously described.^14^ Briefly, a solution of ACh-*d_9_* (0.367 μΜ) in 50% acetonitrile and 0.2% trifluoroacetic acid (TFA) was applied with the TM-sprayer (90 °C, 6 passes, solvent flow rate of 70 μL/min, spray head velocity of 1100 mm/min, and track spacing of 2.0 mm) before the matrix application. The addition of TFA in the solution of the internal standard prevented the enzymatic degradation of ACh. CHCA-*d_4_* (5 mg/mL dissolved in 50% acetonitrile and 0.2% TFA) was applied using the same method as for the internal standard application.

### MALDI Mass Spectrometry Imaging

Tissue sections were imaged in positive ionization mode using a MALDI-FTICR (Solarix XR 7T-2Ω, Bruker Daltonics, Germany) instrument equipped with a Smartbeam II 2 kHz Nd:YAG laser. The laser power was optimized at the start of each analysis. The small laser setting was used to achieve a spatial resolution of 50–100 μm. Red phosphorus was used for external calibration of the methods. Spectra were collected by summing signals from 100 laser shots per pixel. The quadrupole isolation mass-to-charge (*m/z*) ratio (Q1) was set at *m/z* 375, and data were collected over the *m/z* 150–1500 range. The time-of-flight (TOF) and transfer optics frequency values were adjusted to 0.700 ms and 4 MHz, respectively. Two matrix-derived peaks at *m/z* 555.223103 and *m/z* 462.185278 were used as lock masses for internal *m/z* calibration. Samples were analyzed randomized in order to prevent bias due to possible matrix vacuum instability or changes in the mass spectrometer’s sensitivity.

For MALDI-MSI of ACh, the [M] + ion of ACh-*d_9_* (*m*/*z* 155.1740) was used as a lock mass. Continuous accumulation of selected ions (CASI) was used to improve the limit of detection toward the analyte. The Q1 mass was set at *m*/*z* 150, and a mass window of 40 Da was selected to include the endogenous ACh and deuterated analogues. The TOF and frequency values were adjusted to 0.450 ms and 4 MHz, respectively.

### Identification of Neurotransmitters and Metabolites

Chemical structures of the investigated analytes are presented in Figure S1, and both theoretical and empirically determined *m/z* values of the molecular species examined in the study are shown in Table S1. The investigated neurotransmitters and metabolites were identified by exact mass matching with a mass tolerance ±1.2 ppm. In addition, their anatomical distribution and order of derivatization, that is, the number of attached FMP-10 molecules, were used for confirmation, as previously described.^28,30^ When it was possible to isolate the parent ion directly on tissue, MS/MS spectra were obtained and compared to spectra of FMP-10 derivatized standard compounds. For MS/MS, a 1 Da isolation window was used, and different collision energy voltages were applied (from 20 to 40 V).

### MS Image Analysis

MSI data were visualized using flexImaging (v. 5.0, Bruker Daltonics). For further analysis, data were imported to SCiLS Lab (v. 2019c Pro, Bruker Daltonics), and brain regions were annotated according to an anatomic reference atlas.^29^ The annotated Th region included the ventral and lateral groups of the dorsal thalamus (which are related to the sensory-motor and polymodal association cortex), except the midline areas (including PVT) and the medial areas. The data were normalized to the root-mean-square (RMS) of all data points, and all investigated compounds were annotated manually. The area under the curve (AUC) value for each metabolite in the average mass spectra of each brain region was exported from SCiLS for statistical analysis. For all datasets, the exported AUC values were log-transformed and combined in a matrix consisting of 16 observations (four groups of four samples) and 195 variables, that is, 13 metabolites in 16 brain areas (Table S2), for further statistical analysis.

### Statistical Analysis

Multivariate analysis was performed using SIMCA v.13.0 (Sartorius Stedim Biotech, Umeå, Sweden). As all the included variables were of the same type, that is, log-transformed ion intensities, the SIMCA default scaling option of centering and autoscaling to unit variance was considered adequate. Principal component analysis (PCA) was initially conducted to obtain an overview of the data and identify possible outliers (using the Hotelling T2 ellipse, DCrtit, and the distance to model DModX, with 95% confidence intervals). Subsequently, PLS-DA was applied to detect specific neurochemical differences among the four groups. The numbers of components and original variables (*X*) included in the model were defined after evaluating the fit in terms of *R*^2^ and *Q*^2^ values and classification performance, that is, the significance of group separation in the score plot. The variable selection process was based on the variable influence on projection (VIP), which represents the influence of every model variable on the response in each component and the loading value of every variable. Terms with VIP > 1 were considered the most relevant for explaining the dependent variable, that is, classes. Permutation tests (with 100 permutations) were used for validation (Table S3). The significance of PLS-DA components was evaluated using one-way ANOVA with Tukey’s post hoc test (Table S4).

Two-way ANOVA with the FDR adjustment of *P* values was used to analyze the effects of age and tacrine using the online MetaboAnalyst platform version 4.0.^31^ A summary of the F-statistics and *P* values is provided in Table S5. A one-sample *t*-test was used to confirm loading values significantly different than zero. Statistical illustrations were prepared with GraphPad Prism 7.05 (GraphPad Software, San Diego, California USA).

Turnover ratios were calculated using log-transformed values of RMS-normalized ion intensities of the end brain metabolites, that is, HVA, MOPEG, and 5-HIAA, divided by those for the neurotransmitters, that is, DA, NE, and 5-HT, respectively (Table S6).

## Supporting Information Available

Chemical structures of investigated neurotransmitters and metabolites, brain distribution of monoaminergic neurotransmitters, PCA evaluations, theoretical and experimental *m/z* values of the derivatized compounds and corresponding mass accuracy, PLS-DA model validation, two-way ANOVA of the effects of age and tacrine, interaction effects, and additional materials and methods. (PDF)
