# Supplementary material for: Region-Specific and Age-Dependent Multitarget Effects of Acetylcholinesterase Inhibitor Tacrine on Comprehensive Neurotransmitter Systems
Source: ACS Chem Biol. 2021 Dec 21;17(1):147–58. doi: 10.1021/acschembio.1c00803 (PMC8787750; doi:10.1021/acschembio.1c00803)
Supplement: Supplementary file 1 — cb1c00803_si_001.pdf [file cb1c00803_si_001.pdf]

# Supporting Information

## Region-specific and age-dependent multitarget effects of the acetylcholinesterase inhibitor tacrine in comprehensive neurotransmitter systems

**Elva Fridjonsdottir[1a], Theodosia Vallianatou[1a], Ioannis Mantas[2], Reza Shariatgorji[1,3], Anna Nilsson[1,3], Luke S. Schembri[4], Luke R. Odell[4], Per Svenningsson[2], Per E. Andrén[1,3]*.**

[1] Department of Pharmaceutical Biosciences, Medical Mass Spectrometry Imaging, Uppsala University, SE75124 Uppsala, Sweden. Email: per.andren@farmbio.uu.se

[2] Department of Clinical Neuroscience, Section of Neurology, Karolinska Institutet, SE-17177 Stockholm, Sweden

[3] Science for Life Laboratory, Spatial Mass Spectrometry, Uppsala University, SE-75124 Uppsala, Sweden

[4] Department of Medicinal Chemistry, Uppsala University, SE-75123 Uppsala, Sweden

[a] These authors contributed equally. * corresponding author

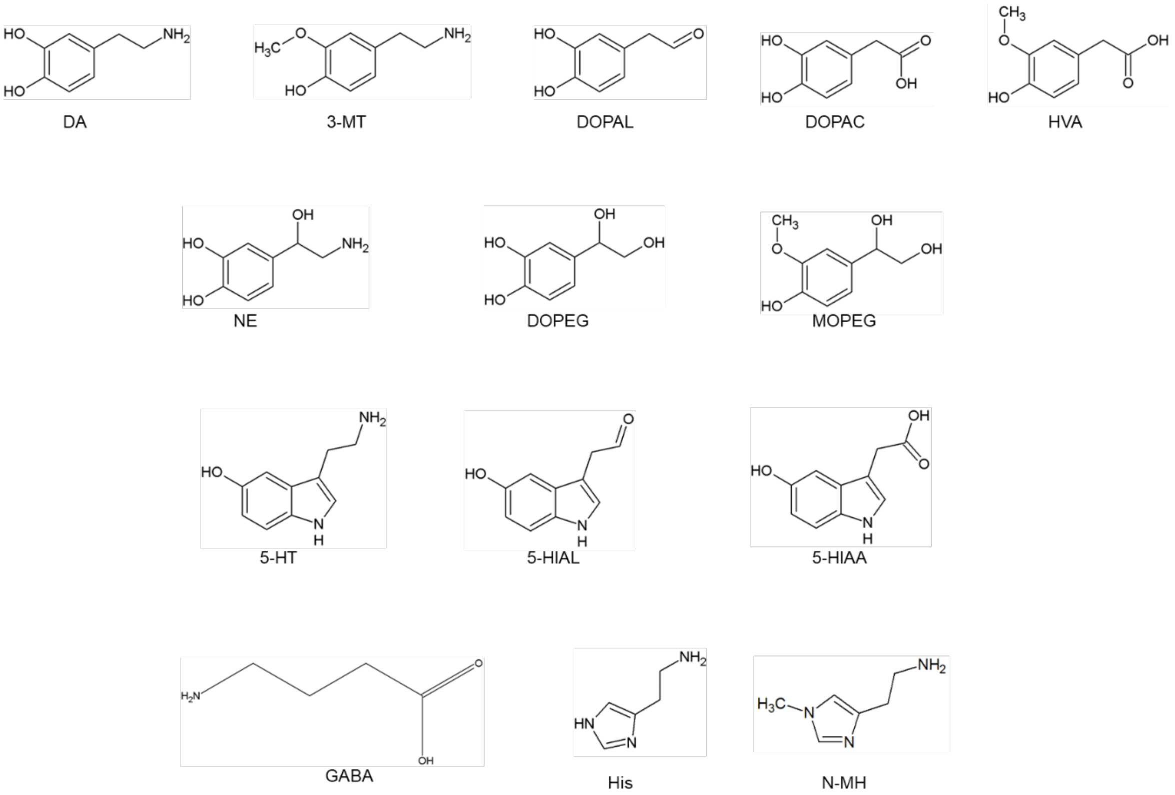


**Figure S1. Chemical structures of investigated neurotransmitters and metabolites.**

Abbreviations: DA, dopamine; 3-MT, 3-methoxy tyramine; DOPAL, 3,4-dihydroxyphenylacetaldehyde; DOPAC, 3,4-dihydroxyphenylacetic acid; HVA, homovanillic acid; NE, norepinephrine; DOPEG, 3,4-dihydroxyphenylglycol; MOPEG, 3-methoxy-4-hydroxyphenylglycol; 5-HT, 5-hydroxy tryptamine; 5-HIAL, 5-hydroxyindole acetaldehyde; 5-HIAA, 5-hydroxyindoleacetic acid; GABA, γ-aminobutyric acid; His, histamine; N-MH, *N*-methylhistamine. N-MH was not included in the statistical analysis due to its low intensity levels.

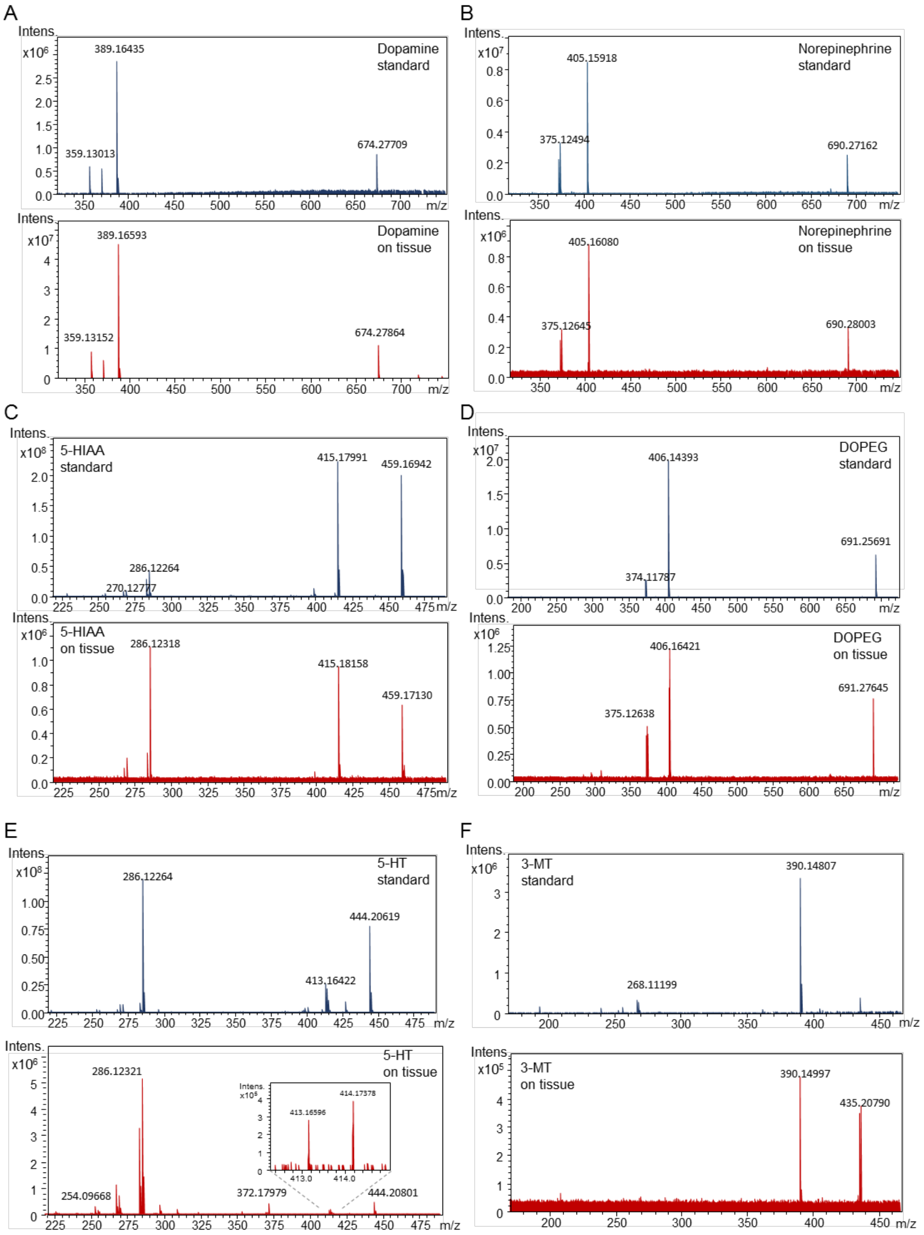


**Figure S2. MS/MS spectra of identified metabolites.**

Comparison of MS/MS spectra collected from standards (blue) with MS/MS spectra obtained from tissue (red). **A,** DA, Q1 mass at *m/z* 674.28, **B,** NE, Q1 mass at *m/z* 690.00, **C,** 5-HIAA, Q1 mass at *m/z* 459.17, **D,** DOPEG, Q1 mass at *m/z* 691.25, **E,** 5-HT, Q1 mass at *m/z* 444.50, **F,** 3-MT, Q1 mass at *m/z* 435.20. For all MS/MS spectra, a collision energy voltage of 30 V was used, except for 3-MT on tissue, where 25 V was used. The isolation window was 1 Da for all parent molecules. Abbreviations: 3-MT, 3-methoxy tyramine; DA, dopamine; DOPEG, 3,4-dihydroxyphenylglycol, 5-HT, 5-hydroxy tryptamine; 5-HIAA, 5-hydroxyindoleacetic acid; Q1 mass, quadrupole isolation *m/z* value.

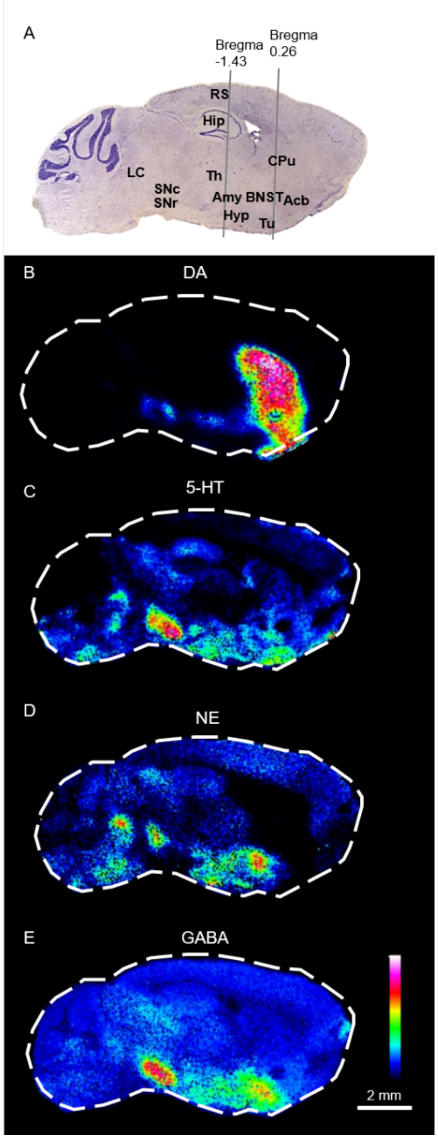


**Figure S3. Brain distribution of monoaminergic neurotransmitters in a sagittal brain tissue section from a 14-m control mouse.**

**A,** Nissl-stained sagittal section (lateral 1.7 mm) from a 14-m control mouse. Brain regions involved in monoaminergic neurotransmission are annotated. The two gray lines indicate coronal levels that were subsequently imaged with MALDI-MSI and analyzed by MVA. **B-E,** MALDI-MS images of DA, 5-HT, NE and GABA, respectively, at a lateral resolution of 60 µm. The color intensity range is scaled to 60% of the maximum intensity. Abbreviations: Acb, nucleus accumbens; Amy, amygdala; BNST, bed nucleus of stria terminalis; CPu, caudate-putamen; DA, dopamine; GABA, γ-aminobutyric acid; Hip, hippocampus; 5-HT, 5-hydroxytryptamine; Hyp, hypothalamus; LC, locus coeruleus; NE, norepinephrine; RSC, retrosplenial cortex; SNc, substantia nigra pars compacta; SNr, substantia nigra pars reticulata; Th, thalamus; Tu, olfactory tubercle.

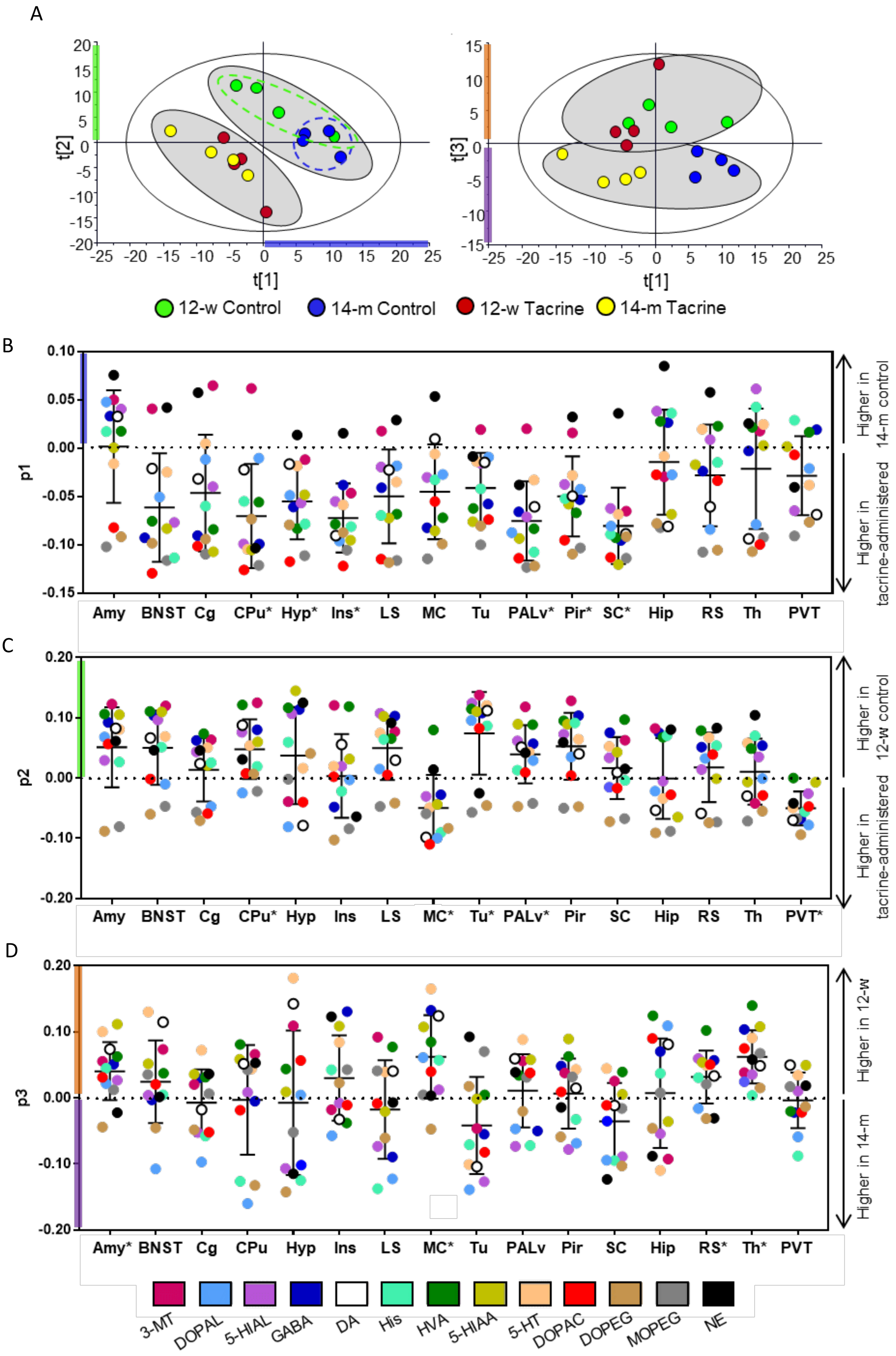
A
t[1]
t[2]
t[3]
12-w Control
14-m Control
12-w Tacrine
14-m Tacrine
B
p1
Amy BNST Cg CPu* Hyp* Ins* LS MC Tu PALv* Pir* SC* Hip RS Th PVT
Higher in 14-m control
Higher in tacrine-administered
C
p2
Amy BNST Cg CPu* Hyp Ins LS MC* Tu* PALv* Pir SC Hip RS Th PVT*
Higher in 12-w control
Higher in tacrine-administered
D
p3
Amy* BNST Cg CPu Hyp Ins LS MC* Tu PALv Pir SC Hip RS* Th* PVT
Higher in 12-w
Higher in 14-m
3-MT DOPAL 5-HIAL GABA DA His HVA 5-HIAA 5-HT DOPAC DOPEG MOPEG NE

**Figure S4. PCA by evaluation of the loadings for each metabolite on the three components of the model.**

**A,** PCA score plots showing grouping of animals according to age and tacrine treatment. **B-D,** Dot plots of the corresponding loading values (p1-p3) according to brain regions. Dots are colored according to neurotransmitter or metabolite species. Brain regions with loading values significantly different from zero (one sample *t*-test) are annotated, **P*<0.05. In A, positive *t*[1] values are highlighted in blue, corresponding to positive p1 values highlighted in **B**, whereas positive *t*[2] values are highlighted in green, corresponding to positive p2 values highlighted in **C**. In **A**, positive *t*[3] values are highlighted in orange and negative *t*[3] values in purple, corresponding to positive and negative p3 values highlighted in **D**. The loading plots derived from PCA reveal how the original variables, i.e., the metabolites in the investigated brain regions, correlated with each other and contributed to the model, with points having high absolute values showing a strong impact on the model, whereas points with values close to zero having a weaker influence. Therefore, considering that the effects of age and tacrine administration were manifested in the score plots, the corresponding loading values of every component (p1-p3) were plotted according to the brain regions to obtain a comparative overview of the role of the metabolites and brain areas. Positive *t*[1] values (highlighted in blue on the *t*[1] axis) were obtained for all the 14-m control samples (annotated by a blue dashed circle), leading to significant separation from tacrine-treated animals of both ages (*P*<0.05), located predominantly on the negative side of *t*[1]. Consequently, the brain regions CPu, Hyp, insular cortex (Ins), PALv, piriform cortex (Pir) and somatosensory cortex (SC) showed a general trend of increased metabolite levels in both tacrine-administered groups compared to the 14-m control group but not the 12-w control group since the latter group was not separated from the tacrine administered groups by *t*[1]. The 12-w control samples (annotated by a green dashed circle) were separated from the tacrine-administered samples according to *t*[2] (highlighted in green on the *t*[2] axis). Examining the p2 values, striatal areas (CPu, PALv and Tu, Fig. 2e) had on average positive values. Therefore, these regions had reduced neurotransmitter and metabolite levels after tacrine administration. The opposite trend was observed for the primary and secondary motor cortex (MC) and periventricular thalamic nucleus (PVT). *t*[3] discriminated between the two age groups, with the amygdala (Amy), MC, RSC and Th having on average highly positive p3 values, indicating increased overall metabolite levels in the 12-w compared to the 14-m samples. Individual metabolites were color coded to investigate general trends associated with the principal components. NE and 3-MT were higher in the 14-m control than the tacrine-administered animals in both age groups, indicated by high p1 values. DOPEG and 3-methoxy-4-hydroxyphenylglycol (MOPEG) were higher in the tacrine-administered animals than in the 12-w control and were located close to each other with low p2 values. The MAO formed monoaminergic metabolites (3,4-dihydroxyphenylacetaldehyde (DOPAL), DOPEG, 5-HIAL) exhibited generally negative p3 values in brain regions relevant to the corresponding system, suggesting age-induced elevation in these metabolites.

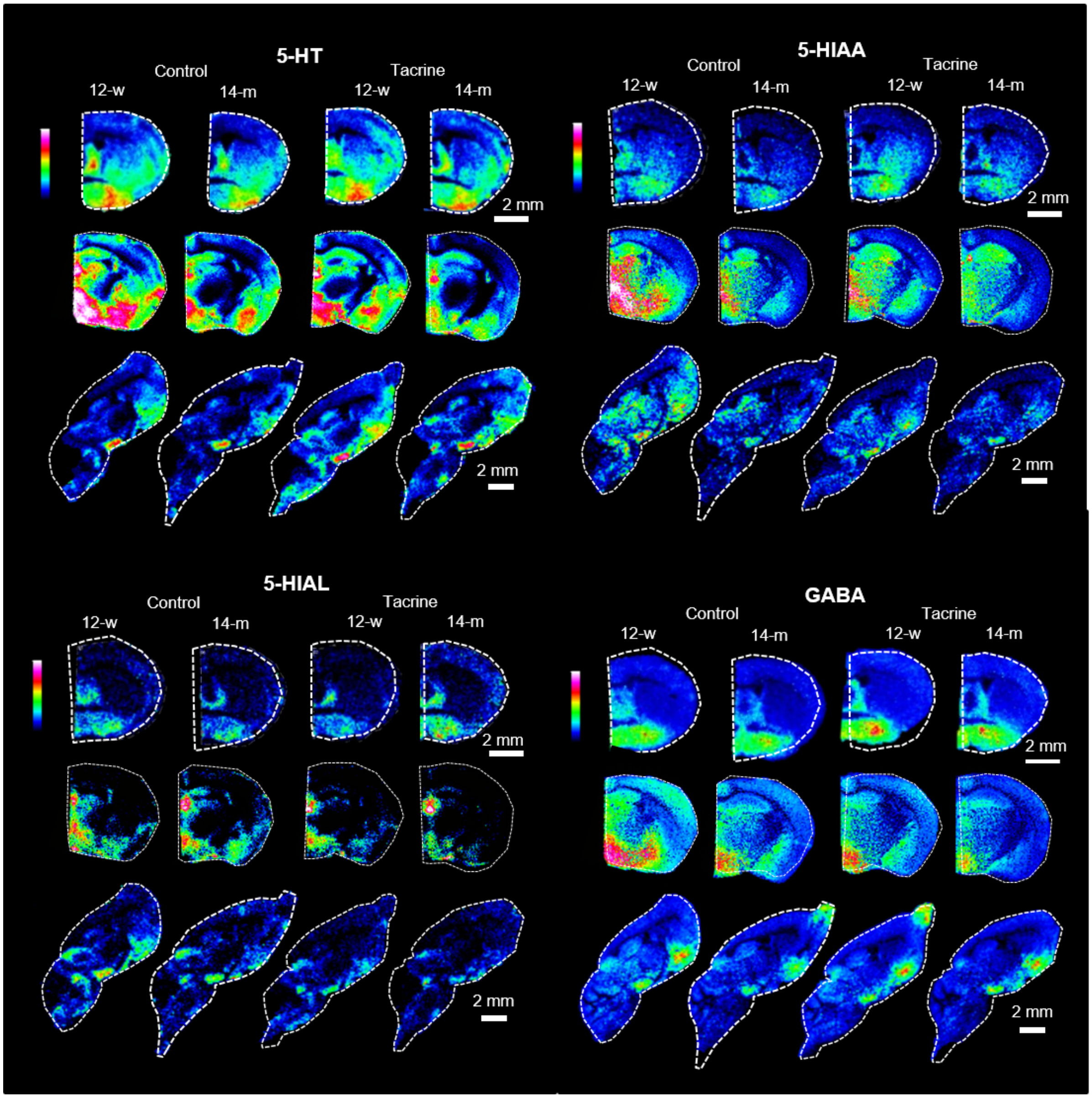


**Figure S5. MALDI-MS imaging of 5-HT, 5-HIAA, 5-HIAL and GABA.**

Two coronal levels are shown, i.e., at 0.20 mm from bregma (upper panel) and -1.43 from bregma (middle panel), and one sagittal (level 1.2 mm from midline). The lateral resolution is 100 µm except for the coronal level at -1.43, for which the lateral resolution is 50 µm. The ion intensity rainbow color scales are scaled according to optimal visualization. Abbreviations: 12-w, 12-week-old; 14-m, 14-month-old; 5-HT, 5-hydroxytryptamine; 5-HIAA, 5-hydroxyindoleacetic acid; 5-HIAL, 5-hydroxyindolealdehyde; GABA, ɣ-aminobutyric acid.

**Table S1. Theoretical and experimental *m/z* values of the derivatized compounds and corresponding mass accuracy.**

| Molecule | Theoretical *m/z* | Experimental *m/z* | Mass error [ppm] |
|---|---|---|---|
| **Cateholamine Pathway** | | | |
| [DA + FMP-10]$^+$ | 421.1910 | 421.1911 | 0.27 |
| [DA + 2 FMP-10]$^+$* | 674.2802 | 674.2794 | -1.18 |
| [3-MT + FMP-10]$^+$* | 435.2067 | 435.2067 | 0.15 |
| [HVA + FMP-10]$^+$* | 450.1699 | 450.1700 | 0.14 |
| [DOPAL + FMP-10]$^+$ | 420.1594 | 420.1595 | 0.33 |
| [DOPAL + 2 FMP-10]$^+$* | 673.2485 | 673.2479 | -0.90 |
| [DOPAC + FMP-10]$^+$ | 436.1543 | 436.1543 | 0.06 |
| [DOPAC + 2 FMP-10]$^+$* | 689.2434 | 689.2427 | -1.13 |
| [NE + FMP-10]$^+$ | 437.1859 | 437.1860 | 0.21 |
| [NE + 2 FMP-10]$^+$* | 690.2751 | 690.2745 | -0.91 |
| [MOPEG + FMP-10]$^+$* | 452.1856 | 452.1855 | -0.19 |
| [DOPEG + FMP-10]$^+$ | 438.1699 | 438.1700 | 0.15 |
| [DOPEG + 2 FMP-10]$^+$* | 691.2591 | 691.2586 | -0.71 |
| **Serotonin Pathway** | | | |
| [5-HT + FMP-10]$^+$* | 444.2070 | 444.2071 | 0.25 |
| [5-HIAA + FMP-10]$^+$* | 459.1703 | 459.1702 | -0.11 |
| [5-HIAL + FMP-10]$^+$* | 443.1754 | 443.1754 | 0.17 |
| **Other Neurotransmitters** | | | |
| [GABA + FMP-10]$^+$ | 371.1754 | 371.1755 | 0.50 |
| [GABA-H2O + FMP-10]$^+$* | 353.1648 | 353.1651 | 0.97 |
| [His + FMP-10]$^+$ | 379.1917 | 379.1918 | 0.37 |
| [His + 2 FMP-10]$^+$* | 632.2808 | 632.2801 | -1.08 |
| [N-MH + FMP-10]$^+$** | 393.2073 | 393.2073 | -0.01 |

* *m/z* value included in statistical analysis. ** The intensity of the metabolite was not high enough for inclusion in the statistical analysis.

**Table S2. Regional mean and standard deviation of area under the curve (AUC) values for all metabolites in the 12-w control, 14-m control, 12-w tacrine and 14-m tacrine groups.**

| | 12-w control | | 14-m control | | 12-w tacrine | | 14-m tacrine | |
|---|---|---|---|---|---|---|---|---|
| Metabolite_Region | Mean | St. dev. | Mean | St. dev. | Mean | St. dev. | Mean | St. dev. |
| 3-MT_AMY | 8.4130 | 5.1101 | 1.2736 | 0.8600 | 0.2484 | 0.1079 | 0.3399 | 0.1159 |
| 3-MT_BNST | 12.1526 | 5.7644 | 4.4650 | 2.3099 | 2.4695 | 0.5750 | 1.7817 | 0.9036 |
| 3-MT_Cg | 0.4294 | 0.3913 | 0.2441 | 0.1594 | 0.1037 | 0.0149 | 0.1277 | 0.0285 |
| 3-MT_CPu | 97.7968 | 12.7912 | 65.0728 | 17.1647 | 36.7910 | 6.6440 | 32.9717 | 7.3309 |
| 3-MT_Hip | 0.0778 | 0.0295 | 0.0919 | 0.0643 | 0.0953 | 0.0453 | 0.1057 | 0.0712 |
| 3-MT_Hy | 1.2343 | 1.4589 | 0.4561 | 0.1793 | 3.0103 | 5.3934 | 0.4120 | 0.2723 |
| 3-MT_INS | 14.4740 | 8.5890 | 3.4701 | 2.1471 | 5.0878 | 3.4783 | 5.3783 | 3.8225 |
| 3-MT_LS | 8.4946 | 5.8383 | 3.8949 | 3.1327 | 2.0987 | 1.7240 | 1.8730 | 1.7088 |
| 3-MT_MC | 0.1172 | 0.0919 | <LOD | | 0.3308 | 0.4356 | 0.0717 | 0.0356 |
| 3-MT_PALv | 21.7253 | 12.7217 | 3.5321 | 2.2671 | 3.3170 | 1.8167 | 2.3797 | 1.7349 |
| 3-MT_Pir | 17.2669 | 15.2144 | 4.5765 | 0.7595 | 2.9782 | 1.2662 | 3.5052 | 2.2982 |
| 3-MT_PVT | <LOD | | 0.3107 | 0.3191 | <LOD | | 0.3086 | 0.5349 |
| 3-MT_RSC | 0.0837 | 0.0850 | 0.0593 | 0.0275 | 0.0978 | 0.1133 | <LOD | |
| 3-MT_SC | 3.1347 | 2.1717 | 1.1716 | 0.8280 | 1.2260 | 0.1575 | 1.4689 | 0.7792 |
| 3-MT_Th | 0.0774 | 0.0476 | 0.0676 | 0.0371 | 0.0794 | 0.0094 | 0.0706 | 0.0210 |
| 3-MT_Tu | 52.7178 | 6.5397 | 26.6712 | 17.8211 | 12.2408 | 10.3652 | 10.1048 | 3.6436 |
| 5-HIAA_AMY | 30.1347 | 4.9561 | 19.4924 | 2.3329 | 21.7671 | 2.4969 | 18.8689 | 2.7499 |
| 5-HIAA_BNST | 74.8817 | 16.5933 | 45.7960 | 4.4543 | 55.6590 | 8.5496 | 63.8000 | 13.4161 |
| 5-HIAA_Cg | 30.7958 | 4.5029 | 19.7728 | 3.8370 | 31.3064 | 4.4983 | 32.5117 | 4.6434 |
| 5-HIAA_CPu | 41.9408 | 7.6521 | 30.2645 | 1.8650 | 42.3492 | 6.9363 | 39.5206 | 6.6413 |
| 5-HIAA_Hip | 23.9848 | 2.2199 | 25.6531 | 7.4299 | 30.7716 | 3.1495 | 31.4497 | 4.4000 |
| 5-HIAA_Hy | 66.0676 | 17.8009 | 43.6464 | 5.6089 | 42.6125 | 9.6426 | 50.0052 | 9.0353 |
| 5-HIAA_INS | 27.1069 | 8.9627 | 17.6630 | 3.0443 | 26.8089 | 4.5132 | 24.8332 | 6.6598 |
| 5-HIAA_LS | 47.1296 | 30.1902 | 34.9263 | 5.7867 | 39.1108 | 13.7274 | 49.9971 | 18.4117 |
| 5-HIAA_MC | 20.2730 | 2.3293 | 13.5748 | 4.0074 | 24.5124 | 6.6884 | 22.5881 | 2.8450 |
| 5-HIAA_PALv | 85.3274 | 15.2006 | 59.6848 | 9.2343 | 75.5610 | 11.9899 | 77.0033 | 12.8034 |
| 5-HIAA_Pir | 32.2662 | 10.3371 | 21.5361 | 3.8773 | 25.8368 | 0.9045 | 25.1497 | 7.1413 |
| 5-HIAA_PVT | 33.3815 | 4.6205 | 28.4337 | 10.9183 | 31.0812 | 4.4895 | 26.6741 | 6.4034 |
| 5-HIAA_RSC | 13.8505 | 3.6800 | 9.9377 | 2.3442 | 12.6091 | 0.8642 | 11.9665 | 5.4957 |
| 5-HIAA_SC | 18.0778 | 5.3073 | 13.6807 | 1.9731 | 18.4283 | 2.4915 | 20.1278 | 4.0358 |
| 5-HIAA_Th | 28.8853 | 3.4489 | 23.2025 | 4.9007 | 29.4572 | 3.2869 | 22.8926 | 2.8992 |
| 5-HIAA_Tu | 49.8377 | 11.7894 | 27.4804 | 7.9472 | 36.5843 | 13.5238 | 40.7802 | 5.2907 |
| 5-HIAL_AMY | 7.4513 | 3.1956 | 9.8401 | 2.9695 | 6.3905 | 3.2054 | 6.0593 | 3.4516 |
| 5-HIAL_BNST | 34.1026 | 15.3733 | 22.9505 | 2.9674 | 23.0362 | 1.5312 | 30.3601 | 5.1812 |
| 5-HIAL_Cg | 7.7227 | 2.2058 | 6.3172 | 4.1997 | 5.1193 | 0.5400 | 8.1536 | 0.9117 |
| 5-HIAL_CPu | 6.7858 | 2.4212 | 5.0419 | 0.7006 | 5.8390 | 1.2752 | 6.7509 | 1.2656 |
| 5-HIAL_Hip | 2.5493 | 1.5988 | 6.9679 | 0.7000 | 2.5287 | 0.8012 | 4.2324 | 2.2934 |
| 5-HIAL_Hy | 37.1563 | 14.2465 | 31.5247 | 3.0093 | 23.7812 | 7.0542 | 40.0539 | 9.8748 |
| 5-HIAL_INS | 15.6844 | 5.5083 | 16.8875 | 6.5198 | 14.5624 | 3.7719 | 17.8296 | 5.3493 |
| 5-HIAL_LS | 24.0912 | 17.8649 | 26.0086 | 3.1230 | 18.5324 | 6.1998 | 28.3620 | 7.2556 |
| 5-HIAL_MC | 6.3513 | 2.3803 | 7.2702 | 2.6211 | 7.6849 | 7.0334 | 9.0466 | 3.5980 |
| 5-HIAL_PALv | 34.2628 | 9.6062 | 34.3240 | 5.1955 | 29.3211 | 4.5166 | 41.2139 | 12.3220 |
| 5-HIAL_Pir | 13.4283 | 4.2870 | 13.1076 | 5.1573 | 9.0045 | 1.4930 | 14.6164 | 5.1213 |
| 5-HIAL_PVT | 19.8674 | 11.1696 | 23.5170 | 4.0101 | 34.8499 | 8.0594 | 33.0658 | 19.4716 |
| 5-HIAL_RSC | 1.6657 | 1.1500 | 2.3390 | 1.4510 | 2.1237 | 1.4492 | 1.2382 | 0.8621 |
| 5-HIAL_SC | 8.1971 | 4.1398 | 9.3154 | 4.6488 | 6.0260 | 0.6745 | 12.2922 | 4.7690 |
| 5-HIAL_Th | 4.2915 | 2.5644 | 4.2929 | 0.9520 | 2.2339 | 0.7388 | 2.1458 | 1.0837 |
| 5-HIAL_Tu | 24.6392 | 7.2820 | 21.7942 | 4.2835 | 11.9741 | 5.7983 | 23.4433 | 4.9364 |
| 5-HT_AMY | 166.8665 | 29.8147 | 129.4203 | 9.3967 | 144.9095 | 10.3030 | 128.5618 | 20.5834 |
| 5-HT_BNST | 247.2645 | 108.5857 | 149.0840 | 37.3342 | 196.9723 | 8.5471 | 160.5743 | 13.1952 |
| 5-HT_Cg | 69.8265 | 18.5775 | 52.7220 | 7.9192 | 55.2495 | 1.7575 | 57.4642 | 12.9258 |

| | | | | | | | | |
|---|---|---|---|---|---|---|---|---|
| 5-HT_CPu | 117.6313 | 13.4381 | 102.0608 | 7.5461 | 117.3080 | 8.4343 | 117.7180 | 8.8734 |
| 5-HT_Hip | 80.3141 | 6.1590 | 86.2397 | 9.2889 | 85.7253 | 10.0248 | 88.5215 | 9.8617 |
| 5-HT_Hy | 243.1693 | 52.9251 | 183.3195 | 27.4900 | 241.4035 | 63.6517 | 189.1295 | 38.4521 |
| 5-HT_INS | 155.3345 | 16.3837 | 149.1183 | 46.8706 | 170.5643 | 9.2623 | 157.4588 | 34.4353 |
| 5-HT_LS | 200.8131 | 150.3902 | 187.6165 | 26.3980 | 203.0545 | 48.2494 | 205.2855 | 44.3112 |
| 5-HT_MC | 73.7616 | 12.3540 | 66.5166 | 12.3569 | 97.3573 | 64.6640 | 65.4069 | 11.0920 |
| 5-HT_PALv | 358.1788 | 14.1920 | 297.8053 | 71.6731 | 350.6578 | 51.3124 | 317.5730 | 42.6148 |
| 5-HT_Pir | 150.5790 | 19.3770 | 140.0048 | 35.1495 | 136.7453 | 4.9332 | 143.1740 | 24.7287 |
| 5-HT_PVT | 201.9055 | 16.4936 | 149.4598 | 7.6365 | 297.9103 | 197.5873 | 224.4880 | 85.7732 |
| 5-HT_RSC | 57.6172 | 11.2776 | 52.6139 | 13.9420 | 49.7385 | 10.3961 | 40.9093 | 14.8750 |
| 5-HT_SC | 91.8121 | 29.3211 | 82.2585 | 8.8734 | 90.9861 | 3.8987 | 89.5495 | 22.2777 |
| 5-HT_Th | 68.1544 | 15.8415 | 43.4250 | 5.9394 | 48.8102 | 5.5193 | 43.7198 | 5.6175 |
| 5-HT_Tu | 412.1513 | 24.3615 | 348.1918 | 105.6513 | 281.8857 | 146.2219 | 351.4913 | 39.3188 |
| DA_AMY | 884.5985 | 442.4307 | 369.2243 | 170.8631 | 324.0108 | 195.0542 | 283.8985 | 111.7224 |
| DA_BNST | 2376.4675 | 581.2956 | 1693.3950 | 419.2145 | 1994.3450 | 373.6335 | 1620.4675 | 297.6852 |
| DA_Cg | 49.1818 | 32.2499 | 55.3879 | 5.2902 | 46.4826 | 11.5880 | 56.9904 | 32.3459 |
| DA_CPu | 15353.4500 | 2173.3912 | 14768.6500 | 882.0285 | 14729.3000 | 689.2161 | 13834.4000 | 1983.1742 |
| DA_Hip | 2.0531 | 0.4953 | 1.1333 | 0.6877 | 3.8450 | 0.1371 | 3.1621 | 1.0156 |
| DA_Hy | 441.4608 | 116.1899 | 361.0215 | 72.7906 | 1301.7815 | 1844.6689 | 436.7283 | 122.9225 |
| DA_INS | 1990.2815 | 1252.8799 | 832.4028 | 565.0364 | 1854.3500 | 1271.9347 | 2013.6950 | 923.0637 |
| DA_LS | 1402.4247 | 409.3926 | 1035.4288 | 470.8855 | 919.3420 | 241.7024 | 1229.5628 | 615.7745 |
| DA_MC | 42.5291 | 17.6021 | 50.1430 | 15.5183 | 218.7933 | 360.2717 | 48.2484 | 12.1422 |
| DA_PALv | 2488.7200 | 1704.0970 | 636.1885 | 37.7751 | 1668.1478 | 748.2039 | 1424.4733 | 735.8550 |
| DA_Pir | 1956.2655 | 1845.0805 | 713.8773 | 273.8857 | 1034.6398 | 493.6299 | 1261.8795 | 322.7695 |
| DA_PVT | 41.5881 | 26.0833 | 12.0668 | 0.6640 | 96.2613 | 65.2235 | 68.7180 | 41.4965 |
| DA_RSC | 3.4413 | 1.2443 | 3.1504 | 1.1941 | 10.6204 | 3.4045 | 4.5234 | 1.3517 |
| DA_SC | 529.9997 | 353.8357 | 343.3095 | 262.4811 | 521.5073 | 158.9662 | 620.8250 | 256.3151 |
| DA_Th | 3.9921 | 1.8058 | 2.1086 | 1.5028 | 6.0119 | 2.6433 | 9.1446 | 9.1742 |
| DA_Tu | 5471.1575 | 1138.3453 | 3900.6850 | 1955.6998 | 3642.8314 | 2497.0695 | 3774.8475 | 498.7439 |
| DOPAC_AMY | 20.4651 | 10.6884 | 10.7159 | 2.8563 | 14.9687 | 2.7138 | 19.0919 | 5.1548 |
| DOPAC_BNST | 106.4012 | 24.1487 | 86.1002 | 9.5863 | 123.7000 | 8.9652 | 131.4768 | 17.8078 |
| DOPAC_Cg | 17.4694 | 4.0116 | 18.6462 | 4.1673 | 25.4314 | 1.4507 | 42.3217 | 15.7079 |
| DOPAC_CPu | 215.1223 | 53.8304 | 196.4578 | 31.9261 | 250.4363 | 29.7056 | 282.5530 | 55.4835 |
| DOPAC_Hip | 3.0847 | 0.5753 | 2.9191 | 1.2907 | 4.0216 | 0.6597 | 3.1464 | 1.1427 |
| DOPAC_Hy | 60.5173 | 16.2336 | 49.3544 | 8.9869 | 78.6084 | 11.2490 | 79.1067 | 7.9761 |
| DOPAC_INS | 63.7715 | 26.8536 | 45.6830 | 14.0354 | 77.5749 | 16.2149 | 91.3520 | 26.7469 |
| DOPAC_LS | 66.1917 | 7.4034 | 50.7036 | 7.0826 | 62.6133 | 7.6560 | 88.0836 | 31.4639 |
| DOPAC_MC | 18.3298 | 2.5483 | 19.7994 | 6.0445 | 30.9441 | 15.5480 | 31.2605 | 8.9861 |
| DOPAC_PALv | 137.7121 | 52.3364 | 87.1017 | 17.9797 | 158.4225 | 23.2718 | 156.9685 | 24.2290 |
| DOPAC_Pir | 71.9316 | 47.2907 | 46.5488 | 7.2736 | 68.9019 | 10.5277 | 81.0509 | 10.9418 |
| DOPAC_PVT | 11.0548 | 3.6778 | 14.5077 | 9.9439 | 16.5874 | 5.7493 | 15.6530 | 6.4774 |
| DOPAC_RSC | 5.1806 | 3.2311 | 4.3441 | 1.0958 | 5.5425 | 2.8850 | 3.9753 | 2.5855 |
| DOPAC_SC | 33.2618 | 11.9137 | 29.3800 | 7.0379 | 42.3771 | 3.8024 | 48.9625 | 13.8469 |
| DOPAC_Th | 5.0259 | 0.6077 | 4.3296 | 0.3436 | 6.7679 | 0.8709 | 6.3323 | 2.4723 |
| DOPAC_Tu | 106.3012 | 36.9495 | 78.7104 | 25.7092 | 98.7029 | 52.2483 | 123.2166 | 20.5746 |
| DOPAL_AMY | 35.9250 | 19.3194 | 30.8307 | 15.5011 | 15.8893 | 3.7747 | 21.1034 | 9.5337 |
| DOPAL_BNST | 138.5635 | 35.1590 | 172.8005 | 14.4878 | 142.4563 | 27.2475 | 206.3528 | 34.4293 |
| DOPAL_Cg | 20.7511 | 4.0505 | 26.2149 | 6.5892 | 20.5864 | 1.3302 | 29.6402 | 4.8928 |
| DOPAL_CPu | 332.8088 | 31.8458 | 579.4278 | 115.1245 | 313.8813 | 39.1813 | 678.6488 | 47.7677 |
| DOPAL_Hip | 4.2328 | 1.3544 | 4.2429 | 2.8036 | 5.7029 | 1.6094 | 4.1294 | 1.5495 |
| DOPAL_Hy | 56.4869 | 8.0677 | 68.6873 | 7.0652 | 77.0343 | 41.8956 | 92.3525 | 20.4430 |
| DOPAL_INS | 71.6814 | 36.5075 | 67.4575 | 45.2967 | 73.9425 | 17.4340 | 128.8948 | 39.1622 |
| DOPAL_LS | 61.4771 | 42.7368 | 97.9842 | 34.2786 | 54.8103 | 10.2279 | 137.9303 | 45.7649 |
| DOPAL_MC | 22.4695 | 3.1922 | 23.3166 | 4.5455 | 29.2758 | 15.2097 | 29.3881 | 5.1679 |
| DOPAL_PALv | 95.7934 | 56.0563 | 61.7997 | 6.7055 | 78.9137 | 20.6349 | 114.6225 | 33.1093 |
| DOPAL_Pir | 66.7309 | 41.3607 | 55.5276 | 21.3202 | 44.6894 | 7.3462 | 74.8544 | 10.7600 |
| DOPAL_PVT | 15.3547 | 8.1838 | 28.0701 | 27.1358 | 29.0203 | 8.4950 | 34.2101 | 8.4584 |

| | | | | | | | | |
|---|---|---|---|---|---|---|---|---|
| DOPAL_RSC | 4.3636 | 1.8363 | 4.0121 | 0.9670 | 5.6654 | 2.2464 | 4.8828 | 0.5949 |
| DOPAL_SC | 29.3352 | 10.5738 | 35.4454 | 14.2477 | 31.6603 | 2.7914 | 53.3727 | 10.4811 |
| DOPAL_Th | 4.9195 | 0.9117 | 5.1654 | 0.8635 | 6.0646 | 0.7674 | 6.3671 | 2.7343 |
| DOPAL_Tu | 163.3383 | 29.5258 | 190.4452 | 79.0648 | 87.7927 | 43.1294 | 200.3620 | 26.5399 |
| DOPEG_AMY | 8.7555 | 0.2826 | 9.5175 | 2.2596 | 17.7071 | 2.2901 | 19.2023 | 4.3321 |
| DOPEG_BNST | 100.1232 | 29.5688 | 122.8490 | 37.2840 | 185.4665 | 15.9290 | 220.9785 | 48.1144 |
| DOPEG_Cg | 33.2369 | 6.9910 | 32.7799 | 12.6608 | 47.5873 | 4.2415 | 67.5979 | 5.0244 |
| DOPEG_CPu | 32.8787 | 11.4973 | 42.2823 | 3.5565 | 36.2973 | 10.0433 | 57.2755 | 11.6192 |
| DOPEG_Hip | 8.5968 | 1.6303 | 10.0359 | 2.4612 | 15.1185 | 2.0518 | 19.3555 | 3.1439 |
| DOPEG_Hy | 91.4205 | 28.4657 | 92.5394 | 20.9883 | 130.6405 | 74.2319 | 197.3875 | 23.5064 |
| DOPEG_INS | 26.5342 | 9.7917 | 31.0575 | 10.5602 | 69.3086 | 40.1171 | 63.1099 | 4.7169 |
| DOPEG_LS | 36.3971 | 2.0755 | 33.8998 | 3.4436 | 51.9038 | 10.6672 | 69.7152 | 14.9511 |
| DOPEG_MC | 27.8540 | 3.0279 | 30.2406 | 9.0668 | 47.2847 | 3.0828 | 67.1668 | 5.9298 |
| DOPEG_PALv | 44.2091 | 17.6026 | 37.6549 | 6.9083 | 69.5361 | 5.8278 | 87.6942 | 11.2007 |
| DOPEG_Pir | 21.5819 | 7.0303 | 21.5149 | 1.8797 | 31.8992 | 3.3977 | 39.9618 | 8.5415 |
| DOPEG_PVT | 31.2178 | 6.0145 | 35.0358 | 20.0114 | 81.9110 | 26.7482 | 81.4734 | 13.2007 |
| DOPEG_RSC | 14.5160 | 5.5378 | 15.3773 | 4.1899 | 27.8923 | 1.6211 | 34.6445 | 8.6873 |
| DOPEG_SC | 22.5507 | 3.3570 | 31.2194 | 8.9516 | 37.1298 | 2.2346 | 59.5551 | 7.6134 |
| DOPEG_Th | 11.0952 | 1.4536 | 9.0897 | 1.6237 | 16.0177 | 1.4314 | 16.1289 | 1.1258 |
| DOPEG_Tu | 20.6417 | 4.6988 | 22.8221 | 9.1968 | 28.2959 | 12.5637 | 34.4697 | 5.7604 |
| GABA_AMY | 468.1855 | 64.2146 | 408.7583 | 83.7700 | 361.4125 | 58.6025 | 361.8048 | 69.4771 |
| GABA_BNST | 905.6423 | 167.8458 | 744.5565 | 97.4500 | 787.4863 | 127.3462 | 916.0375 | 194.8068 |
| GABA_Cg | 363.6013 | 11.8677 | 301.6715 | 31.6216 | 357.6478 | 48.4739 | 351.9380 | 27.6880 |
| GABA_CPu | 336.4188 | 72.7808 | 277.4818 | 12.1335 | 302.2538 | 39.4149 | 340.9020 | 51.6053 |
| GABA_Hip | 355.5453 | 21.1618 | 338.7885 | 22.8498 | 315.3803 | 57.1601 | 304.7993 | 35.1567 |
| GABA_Hy | 941.7475 | 161.2104 | 862.4313 | 81.1537 | 799.2838 | 260.3240 | 994.6220 | 142.5400 |
| GABA_INS | 326.6895 | 43.0835 | 311.9713 | 50.6452 | 407.1335 | 168.4710 | 329.5000 | 21.4657 |
| GABA_LS | 500.9983 | 248.3839 | 526.9590 | 40.7424 | 477.7525 | 189.3361 | 603.6293 | 94.8930 |
| GABA_MC | 333.1368 | 21.1227 | 283.9533 | 45.7638 | 374.0528 | 69.2233 | 340.1595 | 39.1312 |
| GABA_PALv | 1101.4975 | 142.2098 | 1108.5628 | 239.2824 | 1089.9853 | 208.9465 | 1218.5975 | 112.6983 |
| GABA_Pir | 393.2115 | 92.2373 | 332.6198 | 51.0360 | 343.7075 | 45.5985 | 345.5013 | 71.1294 |
| GABA_PVT | 301.0290 | 42.2981 | 308.7708 | 24.2885 | 322.0803 | 84.9293 | 328.6060 | 66.6867 |
| GABA_RSC | 232.7030 | 39.5369 | 206.6853 | 43.3924 | 210.4590 | 59.2400 | 202.4900 | 47.2556 |
| GABA_SC | 344.0055 | 41.2975 | 321.5335 | 43.2307 | 326.6818 | 48.2520 | 375.9508 | 57.6159 |
| GABA_Th | 241.1210 | 31.8000 | 211.6155 | 24.3555 | 220.8160 | 20.1002 | 206.9478 | 20.2879 |
| GABA_Tu | 664.0938 | 169.8527 | 508.9333 | 139.6464 | 527.9870 | 198.9644 | 629.1848 | 35.9661 |
| HIS_AMY | 14.6581 | 2.1763 | 13.8781 | 2.7673 | 13.8758 | 4.1631 | 13.6733 | 1.2827 |
| His_BNST | 38.7774 | 3.5469 | 32.9332 | 1.9383 | 39.1873 | 5.5018 | 41.1040 | 4.3700 |
| His_Cg | 20.6615 | 1.9091 | 17.9871 | 1.8268 | 17.4850 | 0.9657 | 23.4000 | 3.3291 |
| His_CPu | 24.0880 | 1.5123 | 24.9475 | 1.2183 | 22.7366 | 2.1777 | 29.3893 | 1.8299 |
| His_Hip | 11.6156 | 2.1733 | 11.9924 | 3.2841 | 9.5388 | 3.1313 | 9.6808 | 1.6173 |
| His_Hy | 40.3722 | 3.7495 | 38.3511 | 2.9832 | 39.2103 | 8.0798 | 45.9646 | 2.9412 |
| His_INS | 24.9453 | 4.6508 | 24.1794 | 3.4232 | 28.0196 | 6.8662 | 33.0292 | 12.0531 |
| His_LS | 27.6322 | 16.2857 | 34.6752 | 1.4747 | 34.2558 | 6.7848 | 40.3364 | 2.8135 |
| His_MC | 20.0608 | 4.5068 | 19.0715 | 2.5924 | 23.6543 | 7.3209 | 23.4578 | 3.4775 |
| His_PALv | 33.6997 | 3.2956 | 30.1155 | 2.0282 | 32.4057 | 2.8300 | 42.3875 | 5.9298 |
| His_Pir | 25.6512 | 4.1237 | 24.1067 | 4.7561 | 20.3108 | 2.3925 | 29.4318 | 15.2583 |
| His_PVT | 10.6705 | 6.9799 | 13.3113 | 1.3749 | 11.1420 | 6.4039 | 13.4891 | 4.4153 |
| His_RSC | 8.7776 | 1.9638 | 6.5207 | 1.9008 | 6.1928 | 2.7080 | 7.7521 | 0.6891 |
| His_SC | 20.4185 | 2.0968 | 20.7945 | 0.4622 | 20.7005 | 2.3040 | 26.5935 | 3.1624 |
| His_Th | 11.2162 | 2.5404 | 10.1773 | 1.8728 | 8.7648 | 1.5264 | 8.9704 | 1.7174 |
| His_Tu | 34.6098 | 6.6533 | 29.7866 | 7.5598 | 27.2982 | 9.1856 | 37.5421 | 7.9038 |
| HVA_AMY | 30.8724 | 6.6129 | 23.2210 | 5.3675 | 20.0197 | 3.7358 | 20.9361 | 3.0688 |
| HVA_BNST | 73.6352 | 13.8863 | 55.2876 | 7.6346 | 59.1906 | 5.7577 | 66.5726 | 13.1776 |
| HVA_Cg | 52.6402 | 9.8505 | 36.8914 | 4.0292 | 43.0876 | 4.7437 | 59.9491 | 40.7259 |
| HVA_CPu | 123.8875 | 18.8014 | 94.6081 | 10.6608 | 104.1475 | 11.4810 | 100.0451 | 16.5785 |
| HVA_Hip | 22.5113 | 4.9087 | 18.6548 | 3.1768 | 18.9132 | 3.7233 | 14.8146 | 4.0182 |

| | | | | | | | | |
|---|---|---|---|---|---|---|---|---|
| HVA_Hy | 54.0161 | 12.7934 | 40.7426 | 4.7319 | 44.4111 | 4.8595 | 48.3085 | 7.0777 |
| HVA_INS | 64.2740 | 15.9443 | 49.9531 | 8.1195 | 52.5739 | 13.8175 | 61.1850 | 12.8204 |
| HVA_LS | 44.8947 | 27.4670 | 38.7028 | 1.6091 | 43.8306 | 7.7306 | 48.6817 | 13.1757 |
| HVA_MC | 41.9677 | 5.9728 | 31.4715 | 3.4522 | 36.3120 | 0.9393 | 37.6656 | 9.5537 |
| HVA_PALv | 91.3530 | 22.7069 | 67.5596 | 14.0990 | 79.1128 | 13.6074 | 83.6708 | 5.3144 |
| HVA_Pir | 69.4845 | 21.4459 | 50.1068 | 10.3760 | 57.0798 | 3.2849 | 58.3798 | 8.2341 |
| HVA_PVT | 20.1642 | 7.6858 | 17.0714 | 1.7117 | 17.3852 | 5.2188 | 17.5581 | 5.3531 |
| HVA_RSC | 22.4056 | 3.1424 | 16.4905 | 5.0786 | 14.9582 | 2.6806 | 12.7459 | 6.2955 |
| HVA_SC | 50.5787 | 12.0824 | 39.4932 | 2.8797 | 44.2820 | 3.6183 | 47.6293 | 11.7435 |
| HVA_Th | 24.7101 | 6.2001 | 18.8853 | 2.8796 | 21.9808 | 5.6965 | 15.4178 | 3.3459 |
| HVA_Tu | 64.5298 | 21.7203 | 43.5793 | 10.3383 | 49.1541 | 13.4091 | 51.9702 | 1.9894 |
| MOPEG_AMY | 4.5019 | 0.3132 | 3.6383 | 1.1493 | 11.2095 | 1.1159 | 10.1978 | 2.6694 |
| MOPEG_BNST | 21.9611 | 3.8528 | 14.8635 | 4.2860 | 40.5070 | 6.2243 | 38.5702 | 4.8929 |
| MOPEG_Cg | 14.5806 | 2.7821 | 11.0926 | 2.0410 | 23.2813 | 3.1828 | 25.0510 | 4.0046 |
| MOPEG_CPu | 7.3233 | 0.9214 | 5.4740 | 0.8308 | 9.6357 | 0.8745 | 9.1733 | 1.2592 |
| MOPEG_Hip | 3.0538 | 0.7749 | 2.8563 | 1.1489 | 7.8801 | 0.8226 | 7.3450 | 2.4360 |
| MOPEG_Hy | 20.9139 | 3.4097 | 14.8074 | 4.5942 | 37.4056 | 17.9707 | 43.4783 | 6.7837 |
| MOPEG_INS | 10.4993 | 1.1050 | 9.0906 | 2.8501 | 25.8190 | 6.6617 | 22.2382 | 1.8200 |
| MOPEG_LS | 12.5431 | 3.2726 | 8.1591 | 1.6339 | 19.0609 | 3.9085 | 19.5889 | 4.0420 |
| MOPEG_MC | 12.6080 | 0.9303 | 9.2554 | 2.8778 | 21.9140 | 2.7533 | 24.5540 | 3.7990 |
| MOPEG_PALv | 17.6864 | 3.0423 | 12.5052 | 2.2864 | 26.2864 | 1.1682 | 28.3579 | 3.4833 |
| MOPEG_Pir | 10.1263 | 0.6966 | 9.8989 | 3.6014 | 19.6499 | 2.0492 | 16.9387 | 4.2922 |
| MOPEG_PVT | 7.1984 | 4.0349 | 3.1765 | 1.9808 | 16.7092 | 5.9449 | 17.0942 | 8.0690 |
| MOPEG_RSC | 3.2421 | 0.7149 | 2.4479 | 0.6067 | 10.4127 | 2.9440 | 9.6300 | 2.8087 |
| MOPEG_SC | 8.4137 | 0.7215 | 7.8696 | 2.2382 | 17.0189 | 1.9275 | 19.7203 | 2.3168 |
| MOPEG_Th | 3.6114 | 0.8070 | 2.0189 | 0.6051 | 9.2476 | 1.1455 | 5.7756 | 1.2583 |
| MOPEG_Tu | 8.5360 | 2.1782 | 6.6520 | 2.0000 | 14.1209 | 4.4956 | 13.2175 | 2.1103 |
| NE_AMY | 108.8393 | 17.1205 | 116.5933 | 23.2112 | 79.8196 | 13.6496 | 85.5246 | 21.5577 |
| NE_BNST | 1827.3225 | 417.3384 | 2058.7575 | 621.8303 | 1581.5125 | 273.2638 | 1588.1025 | 406.8871 |
| NE_Cg | 303.8078 | 95.2689 | 234.4890 | 37.6395 | 200.5965 | 18.9648 | 223.8838 | 14.3128 |
| NE_CPu | 44.3930 | 14.1821 | 26.5510 | 5.5302 | 42.9621 | 3.2280 | 42.4411 | 8.0785 |
| NE_Hip | 124.4780 | 2.0629 | 141.5880 | 5.4766 | 88.5845 | 6.9672 | 111.5945 | 5.8476 |
| NE_Hy | 1456.4650 | 60.3136 | 1277.7350 | 167.9617 | 708.1047 | 422.3305 | 1027.7925 | 143.3519 |
| NE_INS | 248.0435 | 14.8856 | 269.0313 | 48.8212 | 359.8008 | 316.2575 | 239.2363 | 29.6378 |
| NE_LS | 375.3517 | 102.9220 | 335.7990 | 54.3019 | 256.1400 | 58.1493 | 288.8528 | 86.8258 |
| NE_MC | 277.9393 | 27.5311 | 283.5993 | 46.3677 | 219.6758 | 45.1864 | 263.7200 | 18.4137 |
| NE_PALv | 518.7550 | 140.4986 | 450.0795 | 73.2799 | 470.5898 | 90.6625 | 478.0423 | 84.6290 |
| NE_Pir | 173.3410 | 22.6781 | 190.7565 | 33.5447 | 154.2363 | 16.2007 | 165.1225 | 28.2530 |
| NE_PVT | 621.5358 | 220.0865 | 404.0505 | 197.2317 | 720.6968 | 370.4112 | 738.9105 | 308.1636 |
| NE_RSC | 212.2973 | 46.1333 | 219.4958 | 22.9545 | 156.4730 | 17.8343 | 165.0175 | 23.7239 |
| NE_SC | 203.0175 | 8.2499 | 265.6775 | 40.6724 | 172.8728 | 13.3121 | 242.4323 | 29.8233 |
| NE_Th | 116.3556 | 25.3123 | 73.3891 | 9.7364 | 68.9398 | 13.7354 | 67.5503 | 13.0478 |
| NE_Tu | 117.5665 | 24.6767 | 112.3703 | 18.1072 | 126.1189 | 76.4075 | 115.8725 | 15.8117 |

**Table S3. PLS-DA model validation with permutation tests.**

Permutation tests (100 permutations) were applied by randomly re-ordering the response variables and newly derived $R^2$ and $Q^2$ values were plotted against the degree of correlation between permuted and original data. Models derived after permutation showed considerably inferior statistics ($R^2_{\text{intercept}}$ <<0.5, $Q^2_{\text{intercept}}$ <0.0), indicating the robustness of the original models.

| PLS-DA Model | $R^2_{\text{intercept}}$ | $Q^2_{\text{intercept}}$ |
|---|---|---|
| Class 1 | 0.257 | -0.360 |
| Class 2 | 0.257 | -0.316 |
| Class 3 | 0.248 | -0.425 |
| Class 4 | 0.258 | -0.375 |

**Table S4. Analysis of the significance of PLS-DA components using one-way ANOVA with Tukey's post hoc test.**

| Multiple Comparisons<br>Tukey HSD | | | | | | | |
|---|---|---|---|---|---|---|---|
| | | | | | 95% Confidence Interval | | |
| **Dependent Variable** | | | **Mean Diff.** | ***P*** | **Lower Bound** | **Upper Bound** | ***F*** |
| t[1]-1st PLS-DA component | 12-w Control | 14-m Control | -0.2025 | 0.9659266 | -1.50348 | 1.09854 | 129.443 |
| | | 12-w Tacrine | 5.311 | 0.0000002 | 4.01042 | 6.61244 | |
| | | 14-m Tacrine | 6.566 | <0.0000001 | 5.26461 | 7.86663 | |
| | 14-m Control | 12-w Control | 0.2025 | 0.9659266 | -1.09854 | 1.50348 | |
| | | 12-w Tacrine | 5.514 | 0.0000002 | 4.21289 | 6.81491 | |
| | | 14-m Tacrine | 6.768 | <0.0000001 | 5.46708 | 8.06910 | |
| | 12-w Tacrine | 12-w Control | -5.311 | 0.0000002 | -6.61244 | -4.01042 | |
| | | 14-m Control | -6.768 | 0.0000002 | -6.81491 | -4.21289 | |
| | | 14-m Tacrine | 1.254 | 0.0600879 | -0.04682 | 2.55520 | |
| | 14-m Tacrine | 12-w Control | -6.566 | <0.0000001 | -7.86663 | -5.26461 | |
| | | 14-m Control | -6.768 | <0.0000001 | -8.06910 | -5.46708 | |
| | | 12-w Tacrine | -1.254 | 0.0600879 | -2.55520 | 0.04682 | |
| t[2]-2nd PLS-DA component | 12-w Control | 14-m Control | 4.758 | 0.0000001 | 3.68892 | 5.82697 | 94.101 |
| | | 12-w Tacrine | 0.2701 | 0.8748555 | -0.79890 | 1.33916 | |
| | | 14-m Tacrine | 3.990 | 0.0000006 | 2.92133 | 5.05939 | |
| | 14-m Control | 12-w Control | -4.758 | 0.0000001 | -5.82697 | -3.68892 | |
| | | 12-w Tacrine | -4.488 | 0.0000002 | -5.55684 | -3.41878 | |
| | | 14-m Tacrine | -0.7676 | 0.1981090 | -1.83661 | 0.30145 | |
| | 12-w Tacrine | 12-w Control | -0.2701 | 0.8748555 | -1.33916 | 0.79890 | |
| | | 14-m Control | 4.488 | 0.0000002 | 3.41878 | 5.55684 | |
| | | 14-m Tacrine | 3.720 | 0.0000013 | 2.65120 | 4.78926 | |
| | 14-m Tacrine | 12-w Control | -3.990 | 0.0000006 | -5.05939 | -2.92133 | |
| | | 14-m Control | 0.7676 | 0.1981090 | -0.30145 | 1.83661 | |
| | | 12-w Tacrine | -3.720 | 0.0000013 | -4.78926 | -2.65120 | |
| t[3]-3rd PLS-DA component | 12-w Control | 14-m Control | -2.326 | 0.0085719 | -4.06109 | -0.59030 | 15.883 |
| | | 12-w Tacrine | -3.261 | 0.0005999 | -4.99651 | -1.52572 | |
| | | 14-m Tacrine | -0.04569 | 0.9998185 | -1.78109 | 1.68971 | |
| | 14-m Control | 12-w Control | 2.326 | 0.0085719 | 0.59030 | 4.06109 | |
| | | 12-w Tacrine | -0.9354 | 0.4143416 | -2.67082 | 0.79997 | |
| | | 14-m Tacrine | 2.280 | 0.0098209 | 0.54461 | 4.01540 | |
| | 12-w Tacrine | 12-w Control | 3.261 | 0.0005999 | 1.52572 | 4.99651 | |
| | | 14-m Control | 0.9354 | 0.4143416 | -0.79997 | 2.67082 | |
| | | 14-m Tacrine | 3.215 | 0.0006785 | 1.48003 | 4.95083 | |
| | 14-m Tacrine | 12-w Control | 0.04569 | 0.9998185 | -1.68971 | 1.78109 | |
| | | 14-m Control | -2.280 | 0.0098209 | -4.01540 | -0.54461 | |
| | | 12-w Tacrine | -3.215 | 0.0006785 | -4.95083 | -1.48003 | |

**Table S5. Two-way ANOVA of the effects of age and tacrine, and interaction effects.**

| Two-way ANOVA | | | | | | | | | |
|---|---|---|---|---|---|---|---|---|---|
| **Met_region** | **Age effect** | | | **Tacrine effect** | | | **Interaction effect** | | |
| | *F-value* | *P-value* | *adj. P-value* | *F-value* | *P-value* | *adj. P-value* | *F-value* | *P-value* | *adj. P-value* |
| 3-MT_AMY | 7.2182 | 0.0198 | 0.1913 | 63.9100 | 3.79E-06 | 6.99E-05 | 15.1690 | 0.0021 | 0.1838 |
| 3-MT_BNST | 9.3506 | 0.0099 | 0.1581 | 28.3870 | 1.80E-04 | 0.0017 | 1.5707 | 0.2340 | 0.5107 |
| 3-MT_CPu | 7.7843 | 0.0163 | 0.1780 | 73.8080 | 1.80E-06 | 5.94E-05 | 2.5828 | 0.1340 | 0.4231 |
| 3-MT_PALv | 11.1080 | 0.0060 | 0.1346 | 13.3500 | 0.0033 | 0.0172 | 5.3690 | 0.0390 | 0.4033 |
| 3-MT_Pir | 2.9140 | 0.1135 | 0.3962 | 11.7720 | 0.0050 | 0.0253 | 4.0415 | 0.0674 | 0.4033 |
| 5-HIAA_AMY | 16.6050 | 0.0015 | 0.0447 | 6.2587 | 0.0278 | 0.0971 | 3.9958 | 0.0688 | 0.4033 |
| 5-HIAA_Cg | 6.5634 | 0.0249 | 0.2019 | 10.6400 | 0.0068 | 0.0321 | 9.3628 | 0.0099 | 0.3350 |
| 5-HIAA_MC | 4.7603 | 0.0497 | 0.2729 | 9.7734 | 0.0088 | 0.0380 | 2.6777 | 0.1277 | 0.4231 |
| DA_Hip | 6.1619 | 0.0288 | 0.2019 | 21.4110 | 5.83E-04 | 0.0038 | 1.4812 | 0.2470 | 0.5278 |
| DA_PVT | 5.4080 | 0.0384 | 0.2380 | 16.6620 | 0.0015 | 0.0089 | 1.5324 | 0.2394 | 0.5170 |
| DA_RSC | 6.2587 | 0.0278 | 0.2019 | 17.1150 | 0.0014 | 0.0085 | 4.3639 | 0.0587 | 0.4033 |
| DOPAC_BNST | 0.7864 | 0.3926 | 0.6179 | 14.3720 | 0.0026 | 0.0141 | 2.6191 | 0.1316 | 0.4231 |
| DOPAC_Cg | 4.4071 | 0.0576 | 0.2850 | 22.5240 | 4.75E-04 | 0.0032 | 2.5320 | 0.1375 | 0.4231 |
| DOPAC_Hy | 0.9028 | 0.3608 | 0.6098 | 16.6000 | 0.0015 | 0.0089 | 1.0994 | 0.3151 | 0.6332 |
| DOPAC_INS | 0.2257 | 0.6433 | 0.8180 | 9.2993 | 0.0101 | 0.0427 | 2.1058 | 0.1724 | 0.4287 |
| DOPAC_PALv | 3.5674 | 0.0833 | 0.3311 | 11.2620 | 0.0057 | 0.0283 | 3.2541 | 0.0964 | 0.4231 |
| DOPAC_Th | 1.6411 | 0.2244 | 0.5592 | 10.1180 | 0.0079 | 0.0357 | 0.0335 | 0.8579 | 0.9413 |
| DOPAL_CPu | 103.7900 | 2.93E-07 | 5.94E-05 | 0.7283 | 0.4101 | 0.6073 | 3.1929 | 0.0992 | 0.4231 |
| DOPEG_AMY | 0.4808 | 0.5013 | 0.7217 | 57.5120 | 6.47E-06 | 9.38E-05 | 0.0022 | 0.9636 | 0.9879 |
| DOPEG_BNST | 1.9003 | 0.1932 | 0.5384 | 24.2000 | 3.54E-04 | 0.0027 | 0.0171 | 0.8982 | 0.9448 |
| DOPEG_Cg | 1.6052 | 0.2292 | 0.5592 | 24.2330 | 3.52E-04 | 0.0027 | 3.0596 | 0.1058 | 0.4231 |
| DOPEG_Hip | 4.4401 | 0.0568 | 0.2850 | 44.5450 | 2.28E-05 | 2.80E-04 | 0.2865 | 0.6022 | 0.8278 |
| DOPEG_INS | 0.2219 | 0.6461 | 0.8180 | 22.9280 | 4.42E-04 | 0.0031 | 0.1945 | 0.6670 | 0.8615 |
| DOPEG_MC | 5.3876 | 0.0387 | 0.2380 | 62.1340 | 4.37E-06 | 7.40E-05 | 2.9872 | 0.1095 | 0.4231 |
| DOPEG_PALv | 0.2485 | 0.6271 | 0.8180 | 28.8490 | 1.68E-04 | 0.0016 | 1.6708 | 0.2205 | 0.4865 |
| DOPEG_Pir | 1.4616 | 0.2500 | 0.5592 | 26.6620 | 2.36E-04 | 0.0021 | 0.8553 | 0.3733 | 0.7082 |
| DOPEG_PVT | 0.0015 | 0.9697 | 0.9697 | 24.9360 | 3.13E-04 | 0.0026 | 0.0109 | 0.9188 | 0.9514 |
| DOPEG_RSC | 1.0040 | 0.3361 | 0.6098 | 32.7870 | 9.52E-05 | 0.0010 | 0.1849 | 0.6748 | 0.8615 |
| DOPEG_SC | 18.9840 | 9.33E-04 | 0.0338 | 44.2810 | 2.35E-05 | 2.80E-04 | 0.8844 | 0.3656 | 0.7068 |
| DOPEG_Th | 2.4227 | 0.1456 | 0.4650 | 57.6120 | 6.41E-06 | 9.38E-05 | 2.8691 | 0.1161 | 0.4231 |
| His_CPu | 18.6520 | 0.0010 | 0.0338 | 2.3219 | 0.1535 | 0.3116 | 10.6150 | 0.0069 | 0.2783 |
| His_PALv | 2.3793 | 0.1489 | 0.4650 | 8.9900 | 0.0111 | 0.0460 | 14.1440 | 0.0027 | 0.1838 |
| MOPEG_AMY | 2.8854 | 0.1151 | 0.3962 | 82.6730 | 9.91E-07 | 5.77E-05 | 0.3885 | 0.5448 | 0.8107 |
| MOPEG_BNST | 5.7676 | 0.0334 | 0.2189 | 71.1920 | 2.17E-06 | 5.94E-05 | 3.6608 | 0.0799 | 0.4053 |
| MOPEG_Cg | 1.3520 | 0.2675 | 0.5685 | 56.0440 | 7.37E-06 | 9.97E-05 | 3.9562 | 0.0700 | 0.4033 |
| MOPEG_CPu | 7.0362 | 0.0211 | 0.1945 | 37.0820 | 5.42E-05 | 5.79E-04 | 3.3923 | 0.0903 | 0.4215 |
| MOPEG_Hip | 0.4640 | 0.5087 | 0.7272 | 41.7290 | 3.11E-05 | 3.51E-04 | <0.0001 | 1.0000 | 1.0000 |
| MOPEG_Hy | 0.0432 | 0.8388 | 0.9028 | 15.5020 | 0.0020 | 0.0111 | 2.7548 | 0.1229 | 0.4231 |
| MOPEG_INS | 1.8966 | 0.1936 | 0.5384 | 65.1840 | 3.42E-06 | 6.99E-05 | 0.0604 | 0.8100 | 0.9413 |
| MOPEG_LS | 0.0609 | 0.8093 | 0.8977 | 10.2020 | 0.0077 | 0.0356 | 0.0203 | 0.8891 | 0.9448 |
| MOPEG_MC | 1.4667 | 0.2492 | 0.5592 | 64.5020 | 3.61E-06 | 6.99E-05 | 5.5682 | 0.0361 | 0.4033 |
| MOPEG_ Tu | 1.2634 | 0.2830 | 0.5803 | 20.7670 | 6.58E-04 | 0.0042 | 0.6852 | 0.4240 | 0.7678 |
| MOPEG_PALv | 3.5287 | 0.0848 | 0.3311 | 70.1740 | 2.34E-06 | 5.94E-05 | 8.1055 | 0.0147 | 0.4033 |
| MOPEG_Pir | 0.9913 | 0.3391 | 0.6098 | 24.7350 | 3.23E-04 | 0.0026 | 0.1228 | 0.7321 | 0.8769 |
| MOPEG_PVT | 2.6483 | 0.1296 | 0.4314 | 23.3150 | 4.13E-04 | 0.0030 | 2.1937 | 0.1644 | 0.4287 |
| MOPEG_RSC | 1.6636 | 0.2214 | 0.5592 | 76.5940 | 1.48E-06 | 5.94E-05 | 0.4624 | 0.5094 | 0.8036 |
| MOPEG_SC | 0.1066 | 0.7497 | 0.8617 | 96.3870 | 4.37E-07 | 4.43E-05 | 2.0534 | 0.1774 | 0.4287 |
| MOPEG_Th | 23.1720 | 4.24E-04 | 0.0215 | 80.5630 | 1.14E-06 | 5.77E-05 | 0.2327 | 0.6382 | 0.8468 |
| NE_AMY | 0.3288 | 0.5770 | 0.7757 | 9.7575 | 0.0088 | 0.0380 | 0.0007 | 0.9799 | 0.9946 |
| NE_Hip | 48.7980 | 1.46E-05 | 0.0015 | 127.6900 | 9.42E-08 | 1.91E-05 | 4.0026 | 0.0686 | 0.4033 |
| NE_RSC | 0.3842 | 0.5469 | 0.7472 | 13.5090 | 0.0032 | 0.0170 | <0.0001 | 1.0000 | 1.0000 |
| NE_SC | 31.7280 | 1.10E-04 | 0.0075 | 5.6562 | 0.0349 | 0.1131 | 0.4777 | 0.5026 | 0.8036 |
| NE_Th | 6.3624 | 0.0268 | 0.2019 | 10.6840 | 0.0067 | 0.0321 | 5.2633 | 0.0406 | 0.4033 |

**Table S6. Two-way ANOVA of the effects of age and tacrine, and interaction effects on turnover ratios.**

| Metabolite/transmitter ratio and brain region | Age effect | | | Tacrine effect | | | Interaction effect | | |
|---|---|---|---|---|---|---|---|---|---|
| | *F-value* | *P-value* | *adj. P-value* | *F-value* | *P-value* | *adj. P-value* | *F-value* | *P-value* | *adj. P-value* |
| 5-HIAA/5-HT_AMY | 4.651 | 0.052 | 0.272 | 12.947 | 0.004 | 0.014 | 3.938 | 0.071 | 0.199 |
| 5-HIAA/5-HT_BNST | 1.367 | 0.265 | 0.456 | 7.548 | 0.018 | 0.040 | 13.660 | 0.003 | 0.147 |
| 5-HIAA/5-HT_Cg | 0.867 | 0.370 | 0.555 | 0.860 | 0.372 | 0.525 | 6.772 | 0.023 | 0.173 |
| 5-HIAA/5-HT_Cpu | 16.957 | 0.001 | 0.017 | 1.088 | 0.317 | 0.491 | 9.063 | 0.011 | 0.161 |
| 5-HIAA/5-HT_Hip | 0.017 | 0.897 | 0.957 | 7.585 | 0.017 | 0.040 | 0.085 | 0.775 | 0.865 |
| 5-HIAA/5-HT_Hy | 0.608 | 0.451 | 0.617 | 7.386 | 0.019 | 0.041 | 4.018 | 0.068 | 0.199 |
| 5-HIAA/5-HT_INS | 4.448 | 0.057 | 0.272 | 0.295 | 0.597 | 0.699 | 0.827 | 0.381 | 0.610 |
| 5-HIAA/5-HT_LS | 2.243 | 0.160 | 0.427 | <0.001 | 0.991 | 0.991 | 0.045 | 0.836 | 0.874 |
| 5-HIAA/5-HT_MC | 2.554 | 0.136 | 0.408 | 0.680 | 0.426 | 0.568 | 0.256 | 0.622 | 0.795 |
| 5-HIAA/5-HT_Tu | 0.033 | 0.859 | 0.937 | 0.319 | 0.583 | 0.699 | 8.383 | 0.013 | 0.161 |
| 5-HIAA/5-HT_PALv | 4.032 | 0.068 | 0.295 | 0.764 | 0.399 | 0.548 | 6.216 | 0.028 | 0.173 |
| 5-HIAA/5-HT_Pir | 2.071 | 0.176 | 0.432 | 0.151 | 0.704 | 0.805 | 2.935 | 0.112 | 0.270 |
| 5-HIAA/5-HT_PVT | 6.708 | 0.024 | 0.162 | 2.172 | 0.166 | 0.296 | 0.181 | 0.678 | 0.795 |
| 5-HIAA/5-HT_RSC | 0.002 | 0.968 | 0.969 | 0.438 | 0.521 | 0.641 | 0.040 | 0.845 | 0.874 |
| 5-HIAA/5-HT_SC | 1.304 | 0.276 | 0.456 | 1.823 | 0.202 | 0.346 | 2.314 | 0.154 | 0.313 |
| 5-HIAA/5-HT_Th | 0.497 | 0.494 | 0.625 | 4.672 | 0.052 | 0.099 | 1.512 | 0.242 | 0.431 |
| HVA/DA_AMY | 3.583 | 0.083 | 0.311 | 6.631 | 0.024 | 0.051 | 4.000 | 0.069 | 0.199 |
| HVA/DA_BNST | 0.010 | 0.923 | 0.964 | 5.315 | 0.040 | 0.080 | 4.252 | 0.062 | 0.199 |
| HVA/DA_Cg | 0.069 | 0.797 | 0.911 | 0.894 | 0.363 | 0.525 | 0.442 | 0.519 | 0.731 |
| HVA/DA_Cpu | 2.759 | 0.123 | 0.392 | 3.196 | 0.099 | 0.183 | 2.166 | 0.167 | 0.313 |
| HVA/DA_Hip | 27.430 | <0.001 | 0.009 | 10.569 | 0.007 | 0.024 | 0.878 | 0.367 | 0.608 |
| HVA/DA_Hy | 1.327 | 0.272 | 0.456 | 0.875 | 0.368 | 0.525 | 0.011 | 0.920 | 0.920 |
| HVA/DA_INS | 1.497 | 0.245 | 0.452 | 0.599 | 0.454 | 0.589 | 4.299 | 0.060 | 0.199 |
| HVA/DA_LS | 1.941 | 0.189 | 0.432 | <0.001 | 0.991 | 0.991 | 0.052 | 0.823 | 0.874 |
| HVA/DA_MC | 1.140 | 0.307 | 0.491 | 0.126 | 0.729 | 0.814 | 0.244 | 0.630 | 0.795 |
| HVA/DA_ Tu | 0.466 | 0.508 | 0.625 | 0.076 | 0.788 | 0.840 | 2.263 | 0.158 | 0.313 |
| HVA/DA_PALv | 1.790 | 0.206 | 0.432 | 8.000 | 0.015 | 0.040 | 9.016 | 0.011 | 0.161 |
| HVA/DA_Pir | 0.600 | 0.454 | 0.617 | 0.554 | 0.471 | 0.595 | 4.168 | 0.064 | 0.199 |
| HVA/DA_PVT | 21.764 | 0.001 | 0.009 | 7.795 | 0.016 | 0.040 | 2.560 | 0.136 | 0.310 |
| HVA/DA_RSC | 21.370 | 0.001 | 0.009 | 13.077 | 0.004 | 0.014 | 2.139 | 0.169 | 0.313 |
| HVA/DA_SC | 2.384 | 0.149 | 0.419 | 0.006 | 0.938 | 0.979 | 0.612 | 0.449 | 0.695 |
| HVA/DA_Th | 10.466 | 0.007 | 0.065 | 1.697 | 0.217 | 0.359 | 1.401 | 0.259 | 0.445 |
| MOPEG/NE_AMY | 1.778 | 0.207 | 0.432 | 108.790 | <0.001 | <0.001 | 0.419 | 0.530 | 0.731 |
| MOPEG/NE_BNST | 0.041 | 0.843 | 0.937 | 23.383 | <0.001 | 0.003 | 0.511 | 0.488 | 0.731 |
| MOPEG/NE_Cg | 0.619 | 0.447 | 0.617 | 16.749 | 0.001 | 0.007 | 5.869 | 0.032 | 0.173 |
| MOPEG/NE_CPu | 10.024 | 0.008 | 0.065 | 1.196 | 0.296 | 0.473 | 7.164 | 0.020 | 0.173 |
| MOPEG/NE_Hip | 1.636 | 0.225 | 0.432 | 61.598 | <0.001 | <0.001 | 0.121 | 0.734 | 0.839 |
| MOPEG/NE_Hy | 2.999 | 0.109 | 0.373 | 9.594 | 0.009 | 0.030 | 4.736 | 0.050 | 0.199 |
| MOPEG/NE_INS | 0.576 | 0.463 | 0.617 | 13.559 | 0.003 | 0.014 | 0.195 | 0.667 | 0.795 |
| MOPEG/NE_LS | 1.820 | 0.202 | 0.432 | 0.102 | 0.755 | 0.824 | 0.035 | 0.856 | 0.874 |
| MOPEG/NE_MC | 0.083 | 0.778 | 0.911 | 44.729 | <0.001 | <0.001 | 5.850 | 0.032 | 0.173 |
| MOPEG/NE_ Tu | 0.503 | 0.492 | 0.625 | 7.587 | 0.017 | 0.040 | 0.411 | 0.533 | 0.731 |
| MOPEG/NE_PALv | 0.867 | 0.370 | 0.555 | 17.499 | 0.001 | 0.007 | 4.276 | 0.061 | 0.199 |
| MOPEG/NE_Pir | 0.002 | 0.969 | 0.969 | 25.923 | <0.001 | 0.002 | 0.180 | 0.679 | 0.795 |
| MOPEG/NE_PVT | 4.783 | 0.049 | 0.272 | 7.808 | 0.016 | 0.040 | 3.297 | 0.094 | 0.239 |
| MOPEG/NE_RSC | 0.337 | 0.572 | 0.687 | 51.802 | <0.001 | <0.001 | 0.292 | 0.599 | 0.795 |
| MOPEG/NE_SC | 3.545 | 0.084 | 0.311 | 191.250 | <0.001 | <0.001 | 3.750 | 0.077 | 0.205 |
| MOPEG/NE_Th | 1.684 | 0.219 | 0.432 | 18.188 | 0.001 | 0.007 | 2.202 | 0.164 | 0.313 |
